# Loss of function mutations in the melanocortin 4 receptor in a UK birth cohort

**DOI:** 10.1038/s41591-021-01349-y

**Published:** 2021-05-27

**Authors:** Kaitlin H Wade, Brian YH Lam, Audrey Melvin, Warren Pan, Laura J Corbin, David A Hughes, Kara Rainbow, Jian-Hua Chen, Katie Duckett, Xiaoming Liu, Jacek Mokrosiński, Alexander Mörseburg, Sam Neaves, Alice Williamson, Chen Zhang, I. Sadaf Farooqi, Giles SH Yeo, Nicholas J Timpson, Stephen O’Rahilly

**Affiliations:** 1 Medical Research Council (MRC) Integrative Epidemiology Unit (IEU) at University of Bristol, Bristol, BS8 2BN, UK; 2 Population Health Sciences, Bristol Medical School, University of Bristol, Bristol, BS8 2BN, UK; 3 Wellcome Trust-Medical Research Council Institute of Metabolic Science and NIHR Cambridge Biomedical Research Centre, University of Cambridge, Cambridge CB2 0QQ, UK

## Abstract

Mutations in the melanocortin 4 receptor gene (*MC4R*) are associated with obesity but little is known about the prevalence and impact of such mutations throughout human growth and development. We examined the *MC4R* coding sequence in 5724 participants from the Avon Longitudinal Study of Parents and Children, functionally characterised all non-synonymous *MC4R* variants and examined their association with anthropometric phenotypes from childhood to early adulthood. The frequency of heterozygous loss of function (LoF) mutations in *MC4R* was ~1/337 (0.30%), considerably higher than previous estimates. At age 18 years, mean differences in body weight, body mass index and fat mass between carriers and non-carriers of LoF mutations were 17.76kg (95% CI: 9.41, 26.10), 4.84kg/m^2^ (95% CI: 2.19, 7.49) and 14.78kg (95% CI: 8.56, 20.99), respectively. *MC4R* LoF mutations may be more common than previously reported and carriers of such variants may enter adult life with a substantial burden of excess adiposity.

## Introduction

Mutations disrupting the leptin-melanocortin system have frequently been reported in severe, early-onset human obesity but the prevalence and extent of phenotypic impact of such mutations are unclear. The critical role of the leptin-melanocortin system in the long-term sensing of body fat stores was first established in the 1990s, with defects in this system resulting in obesity in rodents and humans^1–5^. Specifically, the melanocortin 4 receptor (MC4R) is a G-protein coupled, seven-transmembrane receptor expressed widely in the central nervous system^6,7^. The binding of its natural agonists, the pro-opiomelanocortin-derived melanocortin peptides, alpha and beta melanocyte-stimulating hormone (MSH), results in the suppression of food intake and the activation of a subset of autonomic neurons of the sympathetic nervous system^8–10^. Leptin acts on hypothalamic neurons to promote the release of melanocortins and suppress the secretion of the melanocortin antagonist, agouti-related peptide (AGRP)^9,11^.

Severe early-onset human obesity associated with mutations in genes encoding leptin or the leptin receptor are very rare and observed only in individuals homozygous for loss of function (LoF) mutations in those genes^3,5^. However, in the case of mutations in the *MC4R* gene, severe early-onset obesity has been reported in multiple affected members of several families who only carried heterozygote LoF mutations^4,12^. Subsequent studies reported more severe obesity in homozygotes, suggesting a semi-dominant form of inheritance^13^.

Many *MC4R* LoF mutations have been described in obese individuals and families^14–16^ and the severity of disruption of *MC4R* signalling resulting from such mutations has been reported to correlate with adiposity and degree of hyperphagia^17,18^. While early reports based on clinically ascertained cohorts suggested a high penetrance of early-onset obesity, subsequent studies of less highly selected patients demonstrated that the carriage of LoF mutations was not always associated with obesity^15,19^. For example, in a population-based cohort from Germany, Hinney *et al*., using a mutational scanning technique, reported a prevalence of LoF mutations in *MC4R* of ~0.1%^15^. Stutzmann *et al*. reported a prevalence of *MC4R* LoF mutations of 1.7% in obese European adults and that obesity in carriers of the same mutation differed across generations within the same families, providing evidence for gene-environment interaction^19^. In a study of participants in UK Biobank based on high density single nucleotide polymorphism (SNP) genotyping, Turcot *et al*. reported that, while carriers of a rare (0.01%) non-sense mutations were ~7kg heavier, on average, than non-carriers (for an average 1.7m tall individual), the majority of carriers of this mutation were not obese^20^.

Pharmacological agonists of MC4R are in clinical development for the therapy of obesity^21^. In a Phase 1b trial of one such drug, setmelanotide, obese participants carrying heterozygous LoF *MC4R* mutations showed drug-induced weight loss^18^. As preventive efforts for metabolic disease are increasingly focusing on tackling obesity in childhood^22^, knowledge regarding the prevalence of *MC4R* LoF mutations and their impact on body composition and growth during the first decades of life will be increasingly important and relevant to future drug development.

In order to determine the frequency of functionally impaired *MC4R* mutations and their clinical and phenotypic consequences throughout childhood, adolescence and early adult life in an unselected UK population, we examined the *MC4R* coding sequence in 5724 participants from the Avon Longitudinal Study of Parents and Children (ALSPAC), a birth cohort recruited in Bristol (UK) in 1990-92 and repeatedly followed up until early adulthood^23,24^. We functionally characterised all non-synonymous *MC4R* variants via *in vitro* assays and examined the relationship between carriage of LoF mutations and anthropometric variables from childhood to early adulthood.

## Results

### Detection of MC4R mutations by pooled sequencing

**Extended Data Figure 1:**
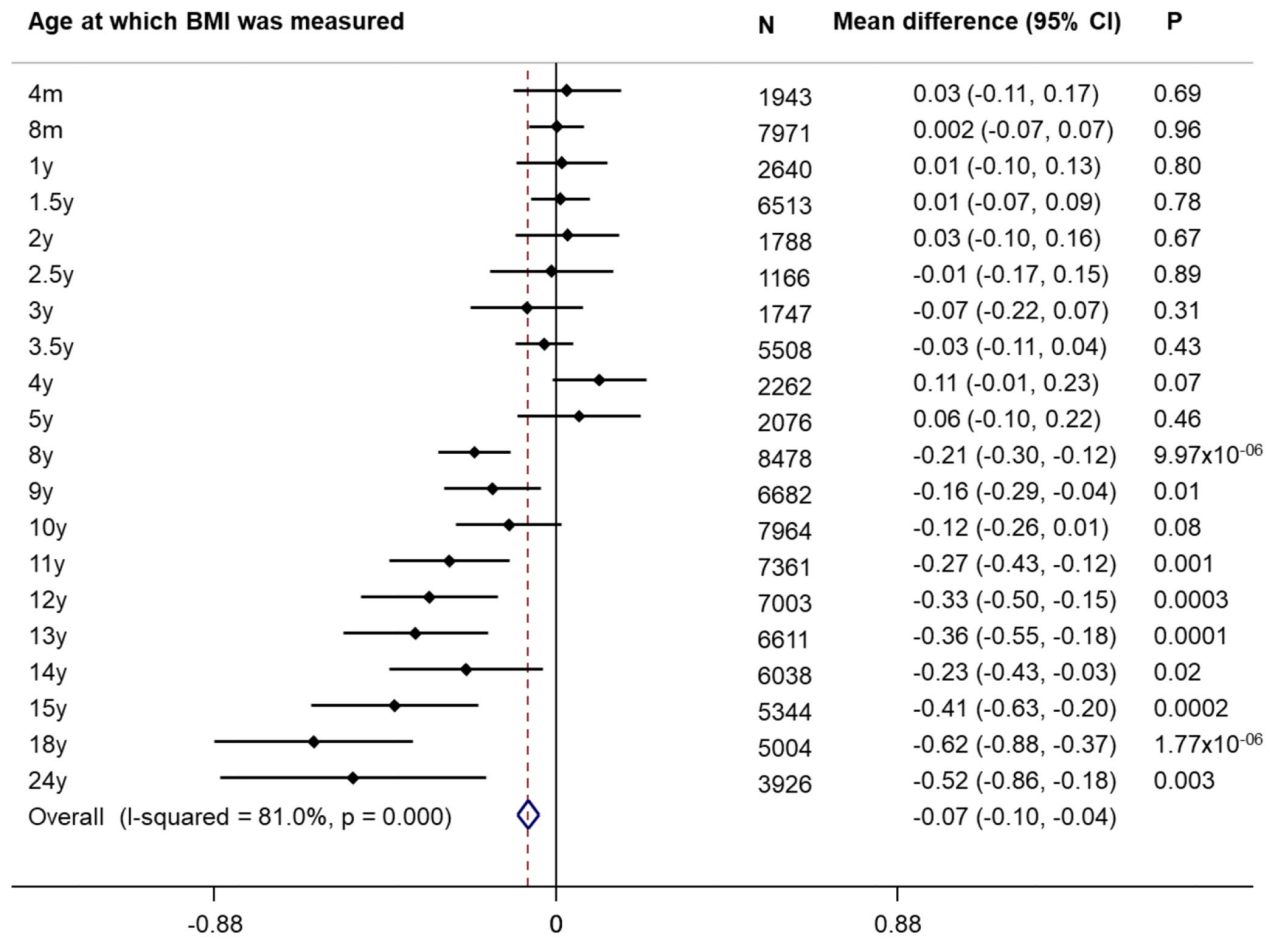
Differences in body mass index in those sequenced for *MC4R* LoF mutations and those not sequenced from the rest of ALSPAC. Estimates reflect the mean difference (95% CI) in BMI (kg/m^2^) comparing those sequenced for MC4R LoF mutations to those not sequenced, obtained from two-sided t-tests (p-values were not corrected for multiple comparisons). ALSPAC = Avon Longitudinal Study of Parents and Children; BMI = body mass index; CI = confidence interval; LoF = loss of function.

ALSPAC is a birth cohort originally comprised of >75% of all pregnancies delivered in the Greater Bristol area from 1990-92. Whilst a specific cohort, ALSPAC represents a population-based sample with deep longitudinal phenotyping suitable for the dissection of *MC4R* mutation associations. Characteristics of the sequenced set of individuals and the complete ALSPAC cohort were similar (Supplementary Table 1, Extended Data Figure 1), suggesting that the sequenced set were at least representative of the wider cohort, which is well described in terms of both demographic profile^23^ and attrition^25^.

The single exon gene, *MC4R*, was sequenced using a novel, cost-effective high-throughput approach, which involved pooling DNA from 5993 unrelated participants of ALSPAC into 120 pools (see Methods). We established that this approach had a sensitivity equivalent to whole exome sequencing (WES) of each individual DNA sample and ~90% specificity in detecting single heterozygous *MC4R* mutations in pools of up to 50 individual DNA samples (see Methods). In total, 29 different non-synonymous mutations in *MC4R* were identified during sequencing of the cohort, including two frameshift/premature stop mutations and 27 missense mutations (Table 1). Two of the missense mutations were the commonly occurring p.V103I and p.I251L variants. Sanger sequencing confirmed the presence of all rare *MC4R* mutations (minor allele frequency (MAF) <0.1%) and that carriers were heterozygous.

### The impact of MC4R mutations on canonical cAMP signalling

MC4R transduces external stimuli through Gα_s_-mediated activation of adenylyl cyclase, resulting in the increase of cytoplasmic levels of cyclic adenosine monophosphate (cAMP). Of the 29 non-synonymous mutations that were detected, 22 had previously been reported in terms of their ability to generate a cAMP response to melanocortin ligands and their association with human obesity (Supplementary Table 2). Of the 22 historically studied variants, two were reported to show complete loss of function (cLoF), nine to have a partial loss of function (pLoF), two to show gain of function (GoF) and nine to show wild-type (WT) like activity (see Methods and Supplementary Table 2 for classification criteria and references).

**Figure 1:**
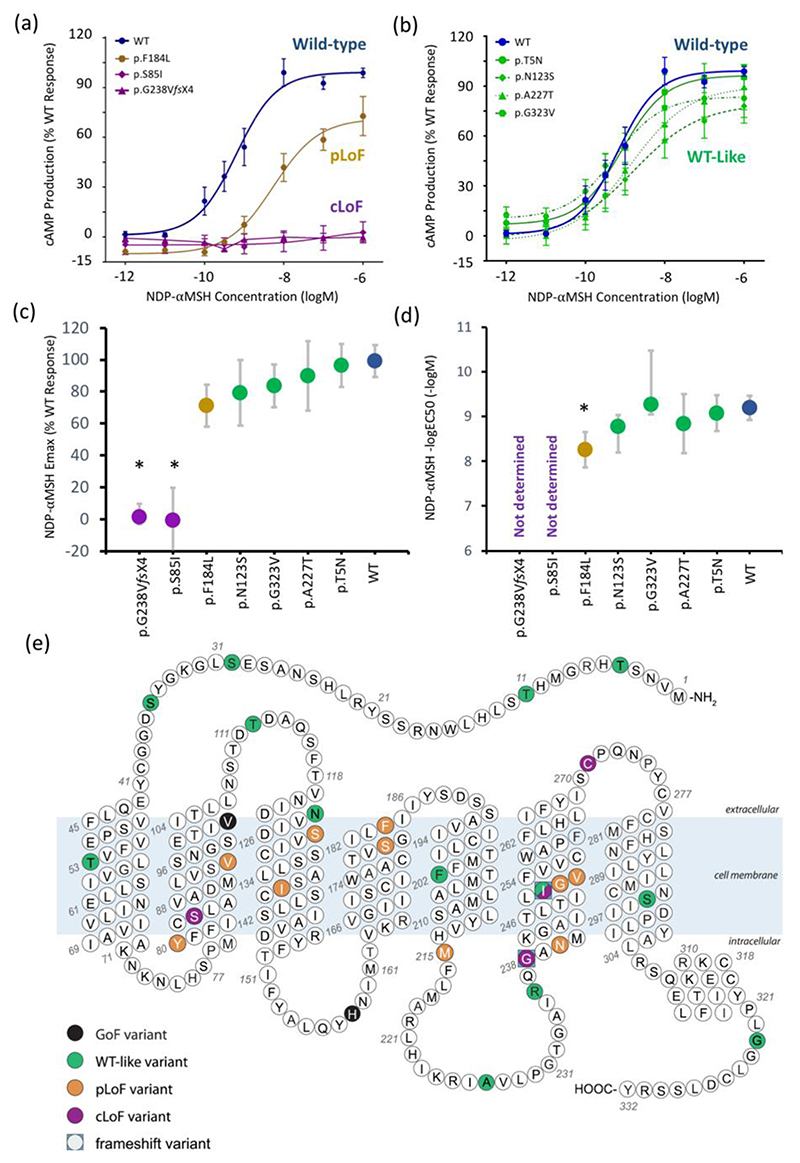
Ligand-activated cAMP accumulation of previously uncharacterized *MC4R* mutations (a) Dose-response characteristics of NDP-αMSH-mediated cAMP production for the previously uncharacterized loss of function mutations found in ALSPAC. Means +/- standard errors are shown (N=3-6, each from an independent experiment); (b) Dose-response characteristics of the previously uncharacterised ‘WT-like’ variants. Means +/- standard errors are shown (N=5-6, each from an independent experiment); (c) Relative maximal efficacy (Emax) of NDP-MSH on MC4R mutants compared to WT, represented as estimated % WT response +/- 95% CI (p-values and sample sizes are listed in Supplementary Table 2); (d) The potency of NDP-MSH (–logEC50) on MC4R mutants, represented as estimated –logEC50 +/- 95% CI (p-values and sample sizes are listed in Supplementary Table 2); and (e) Schematic representation of MC4R showing all 29 mutations identified in the study cohort and their functional classification. There are 2 mutations at the p.I251 residue, p.I251L (WT-like) and p.I251WfsX34 (cLoF). *p<0.05 (p-values were two-sided and not corrected for multiple comparisons). cAMP = cyclic adenosine monophosphate; CI = confidence interval; cLoF = complete loss of function; GoF = gain of function; NDP-αMSH = [Nle4,D-Phe7]-α-melanocyte-stimulating hormone; pLoF = partial loss of function; WT-like = wild-type like; Emax = Relative maximal efficacy; EC50 = Half maximal effective concentration.

We next generated the seven previously uncharacterised mutants by site-directed mutagenesis and, in transiently transfected COS-7 cells, measured cAMP accumulation in response to escalating doses of [Nle^4^,D-Phe^7^]-α-melanocyte-stimulating hormone (NDP-αMSH) (Figure 1). Of the seven variants characterised, two were cLoF mutations (p.S85I and p.G238V*fs*X4) and one was a pLoF mutation (p.F184L). The four remaining variants (i.e., p.T5N, p.N123S, p.A227T and p.G323V) all displayed ‘WT-like’ activity (Figure 1). In total, there were 14 rare *MC4R* LoF mutations (four cLoF and 10 pLoF) identified in the study cohort (Supplementary Table 2, Supplementary Table 3).

**Extended Data Figure 2:**
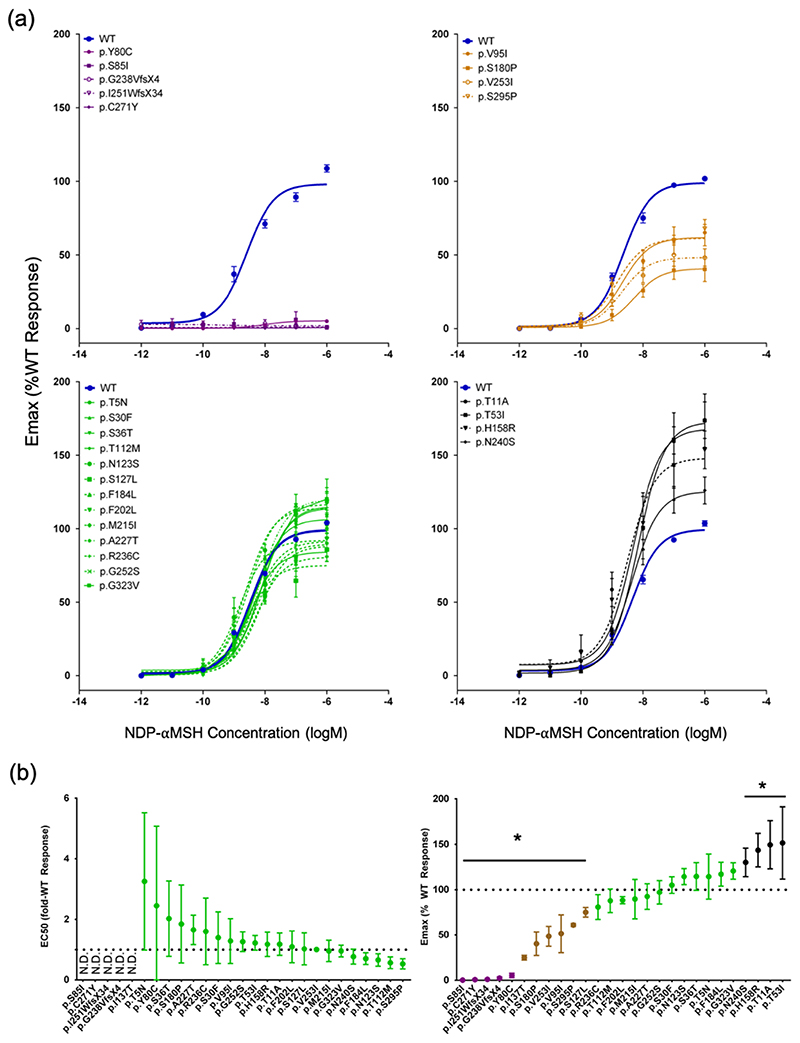
β-arrestin-2 functional classification of *MC4R* variants. Colours represent cLoF (purple), pLoF (light brown), WT-like (green) and GoF (black). β-arrestin-2 coupling activity of MC4R mutations were characterised using a protein-protein interaction assay. (a) Dose response curves of MC4R mutants upon activation by NDP-MSH grouped by cLoF, pLoF, WT-like and GoF compared wild-type MC4R. Means +/- S.E.M. are shown (N=3-8 independent experiments); and (b) The relative EC_50_ (fold WT response, left panel) and E_max_ (% WT response right panel) were determined for each mutants and are presented in mean and 95% CI. * indicates p<0.05 by paired t-test (p-values were two-sided and uncorrected for multiple comparisons). CI = confidence interval; cLoF = complete loss of function; GoF = gain of function; N.D. = not determined; NDP-αMSH = [Nle4,D-Phe7]-α-melanocyte-stimulating hormone; pLoF = partial loss of function; WT-like = wild-type like.

More recently, β-arrestin-2 coupling has been postulated to provide an important alternative post-receptor signal relevant to the control of body weight^26^. We examined NDP-αMSH-induced β-arrestin-2 coupling for all 27 rare variants (the common variants p.V103I and p.I251L were excluded) in transiently transfected HEK-293 cells. Using the same efficacy (E_max_) and potency (EC_50_) based criteria, we found that 10 of the 14 variants that were annotated as LoF for cAMP accumulation also showed impaired β-arrestin-2 coupling (Extended Data Figure 2, Supplementary Table 3). As cAMP is still considered to be the canonical signalling pathway for *MC4R*, our primary analyses of the association between *in vitro* function and clinical phenotype were undertaken using the cAMP-based functional classification with β-arrestin-2-based functional classification as a sensitivity analysis.

### Identification of rare variant carriers

Once we completed the functional characterisation of *MC4R* mutations, we unencrypted the sequenced pools to identify specific individuals carrying these mutations in ALSPAC. Of the 5993 individuals sequenced, a total of 5724 were used in the following analyses characterising the prevalence and downstream effects of *MC4R* LoF mutations on anthropometric traits due to exclusions of duplications and related individuals (see Methods for QC process). Of these 5724 participants, 40 individuals carrying 27 unique variants were confirmed as true positives (see Methods). Of these, 17 individuals carried a heterozygous copy of one of the 14 LoF mutations (Table 2), giving a frequency of 0.30% of LoF mutations (i.e., approximately 1 in 337). Four participants (0.07%) carried a cLoF mutation and 13 (0.23%) carried a pLoF mutation. Twenty-one (0.37%) individuals carried WT-like mutations and two (0.03%) individuals had GoF mutations.

### Age-specific associations with anthropometric traits

**Extended Data Figure 3:**
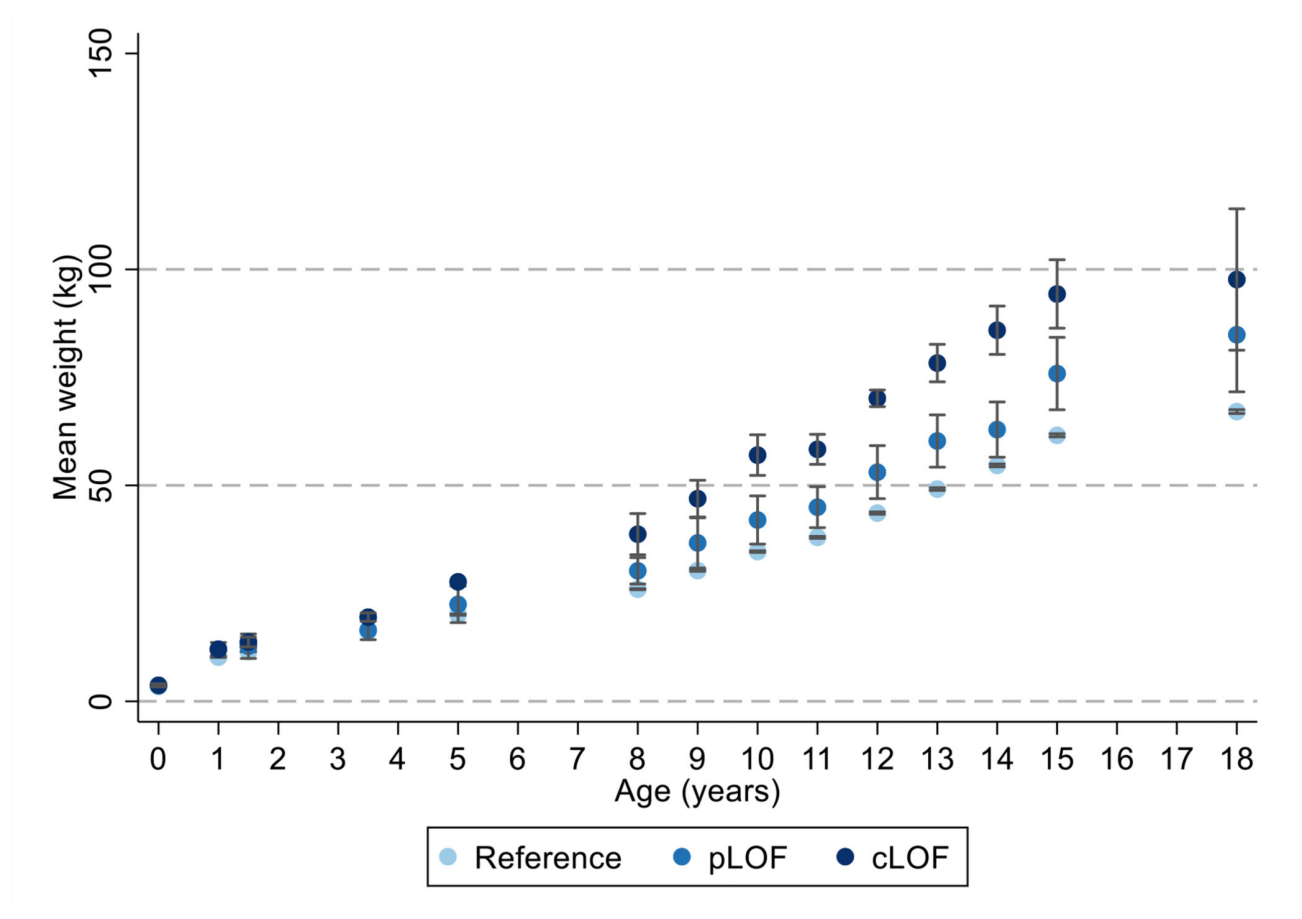
Mean weight across time with *MC4R* LoF of cAMP accumulation. Mean weight +/- 95% CI at different ages (sample sizes across all ages presented in Supplementary Table 4) with MC4R LoF of cAMP (carriers of pLoF and cLoF) and the reference group (i.e., non-LoF carriers – combining individuals with synonymous, common variations or no LoF mutations and individuals with WT-like and GoF mutations). Figures only show results where all mutational groups (i.e., WT-like, GoF, pLoF and cLoF mutations) were represented by at least one individual at all time points between birth and 24 years. CI = confidence interval; cLoF = complete loss of function; GoF = gain of function; LoF = loss of function; pLoF = partial loss of function; WT = wild-type.

**Extended Data Figure 4:**
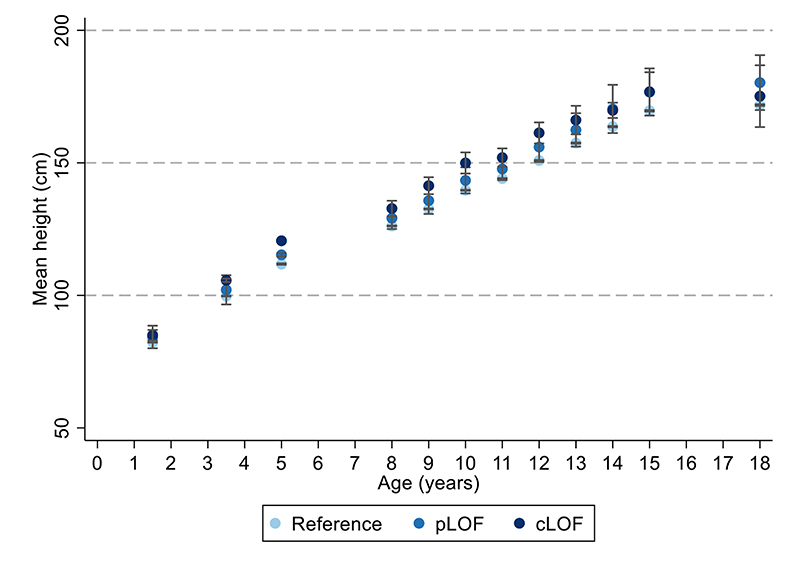
Mean height across time with *MC4R* LoF of cAMP accumulation. Mean height +/- 95% CI at different ages (sample sizes across ages presented in Supplementary Table 4) with MC4R LoF of cAMP (carriers of pLoF and cLoF) and the reference group (i.e., non-LoF carriers – combining individuals with synonymous, common variations or no LoF mutations and individuals with WT-like and GoF mutations). Figures only show results where all mutational groups (i.e., WT-like, GoF, pLoF and cLoF mutations) were represented by at least one individual at all time points between birth and 24 years. CI = confidence interval; cLoF = complete loss of function; GoF = gain of function; LoF = loss of function; pLoF = partial loss of function; WT = wild-type.

**Figure 2:**
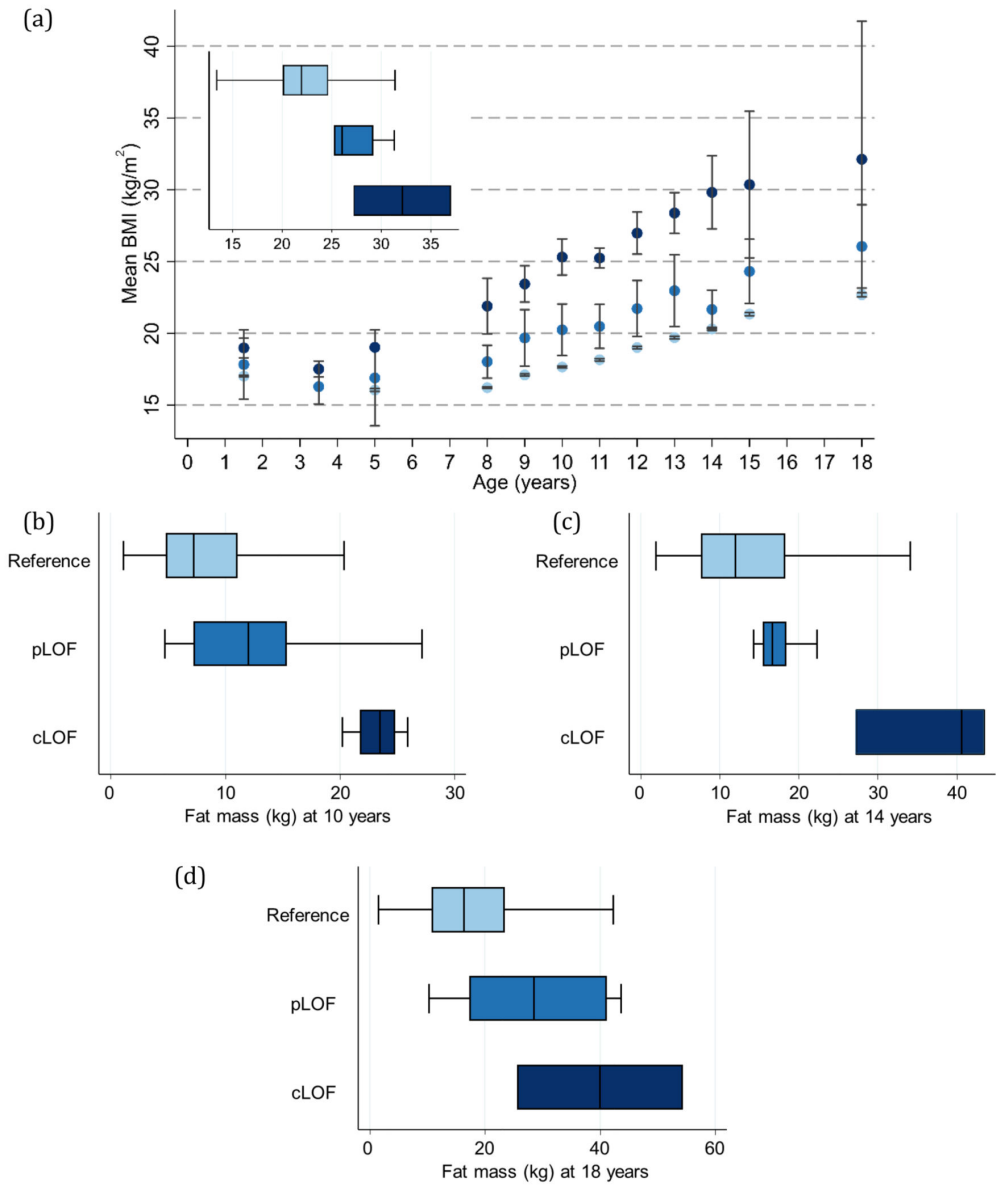
BMI at different ages with MC4R LoF of cAMP accumulation (a) Mean BMI +/- 95% CI at different ages (sample sizes across all ages presented in Supplementary Table 4) and box plot showing distribution (median, IQR and range) of BMI at age 18 years (N=3499); (b-d) box plots showing distribution (median, IQR and range) of the earliest (10 years; N=5109), middle (14 years; N=4295) and last (18 years; N=3408) measure of fat mass with MC4R LoF of cAMP accumulation (carriers of pLoF and cLoF) and the reference group (i.e., non-LoF carriers – combining individuals with synonymous, common variations or no LoF mutation and those with WT-like and GoF mutations). Figures only show results where all LoF mutations (i.e., pLoF and cLoF mutations) were represented by at least one individual at all time points between birth and 24 years. Reference, pLoF and cLoF groups are depicted in light, medium and dark blue, respectively. BMI = body mass index; CI = confidence interval; cLoF = complete loss of function; GoF = gain of function; IQR = interquartile range; LoF = loss of function; pLoF = partial loss of function; WT-like = wild-type like.

Age-specific analyses were conducted using linear regression across all measures of selected anthropometric traits between birth and 24 years. There was a positive association between carriage of *MC4R* LoF mutations and BMI in childhood, adolescence and adulthood, with the mean difference increasing over time from as early as 5 years. This effect was greatest at age 18 years (Supplementary Table 4 and Supplementary Table 5, Figure 2), where the mean difference in BMI between carriers and non-LoF carriers of *MC4R* mutations was 4.84kg/m^2^ (95% CI: 2.19, 7.49; P=3.42x10^-04^). Similarly, there was a positive association between carriage of *MC4R* LoF mutations and weight (Supplementary Table 4, Extended Data Figure 3), with the greatest difference between carriers and non-LoF carriers seen at 18 years (mean difference: 17.76kg; 95% CI: 9.41, 26.10; P=3.11x10^-05^). There was a smaller overall effect of *MC4R* LoF carriage on height over time (Supplementary Table 4, Extended Data Figure 4), with the greatest difference at 12 years (mean difference: 6.53cm; 95% CI: 2.88, 8.54; P=4.50x10^-04^). For both BMI and weight, there was an attenuation of effect of carriage of *MC4R* LoF mutations from age 18 to 24 years; however, it is worth noting that there were no individuals carrying cLoF mutations with anthropometric data at this age.

*MC4R* LoF also showed a positive association with fat mass measured by dual energy x-ray absorptiometry (DXA) (Supplementary Table 6), with the greatest difference between carriers and non-LoF carriers at 18 years (mean difference: 14.78kg; 95% CI: 8.56, 20.99; P=3.27x10^-06^). The positive association between *MC4R* LoF and lean mass was also consistent over time (Supplementary Table 6), with the greatest difference between carriers and non-LoF carriers seen at 12 years (mean difference: 4.28kg; 95% CI: 2.08, 6.48; P=1.38x10^-04^).

Of the four waist-hip ratio (WHR) measures available in ALSPAC, the mean difference in WHR with *MC4R* LoF was the same at ages 10, 12 and 24 years, with carriers of *MC4R* LoF mutations increasing WHR by 0.04 (Supplementary Table 6) compared to non-LoF carriers. This difference was smaller at age 8 years (0.01; 95% CI: -0.01, 0.03; P=0.48) and increased to 0.04 at 24 years (95% CI: -0.03, 0.10; P=0.29); however, there were no individuals carrying a cLoF mutation and a measure of WHR at age 24 years.

In contrast to the associations seen between *MC4R* LoF carriage and anthropometric traits, there were no substantive differences in BMI, weight or height among individuals carrying “WT-like” receptors compared to non-LoF carriers not carrying WT-like mutations (Supplementary Table 7).

**Extended Data Figure 5:**
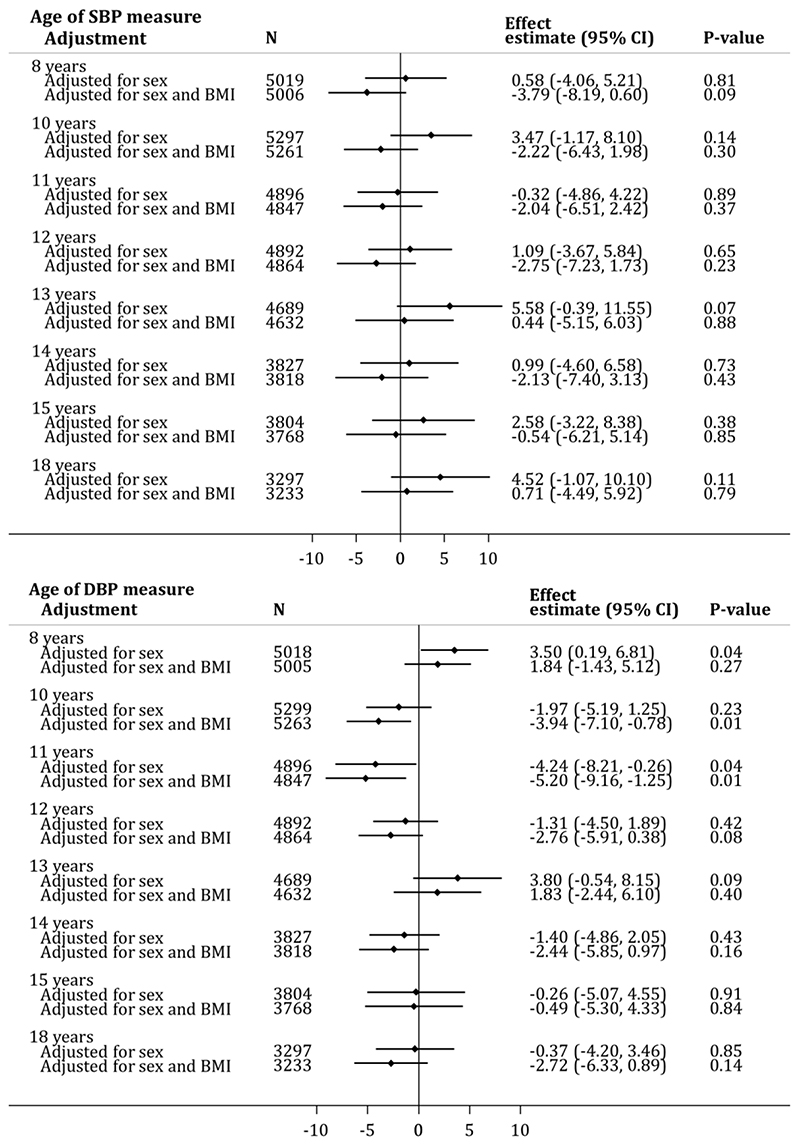
Association between *MC4R* LoF of cAMP accumulation and arterial BP across age in a model adjusting only for sex and a model adjusted for sex and BMI at the same age. Estimates represent the change (mmHg) in SBP or DBP (top and bottom panel, respectively) in carriers (i.e., pLoF or cLoF) vs. non-LoF carriers (i.e., individuals with synonymous, common variations or no LoF mutations and individuals with WT-like and GoF mutations) of MC4R LoF mutations, obtained from linear regression (p-values presented are two-sided and not corrected for multiple comparisons). BMI = body mass index; BP = blood pressure; CI = confidence interval; cLoF = complete loss of function; DBP = diastolic blood pressure; GoF = gain of function; LoF = loss of function; pLoF = partial loss of function; SBP = systolic blood pressure; WT-like = wild-type like.

**Extended Data Figure 6:**
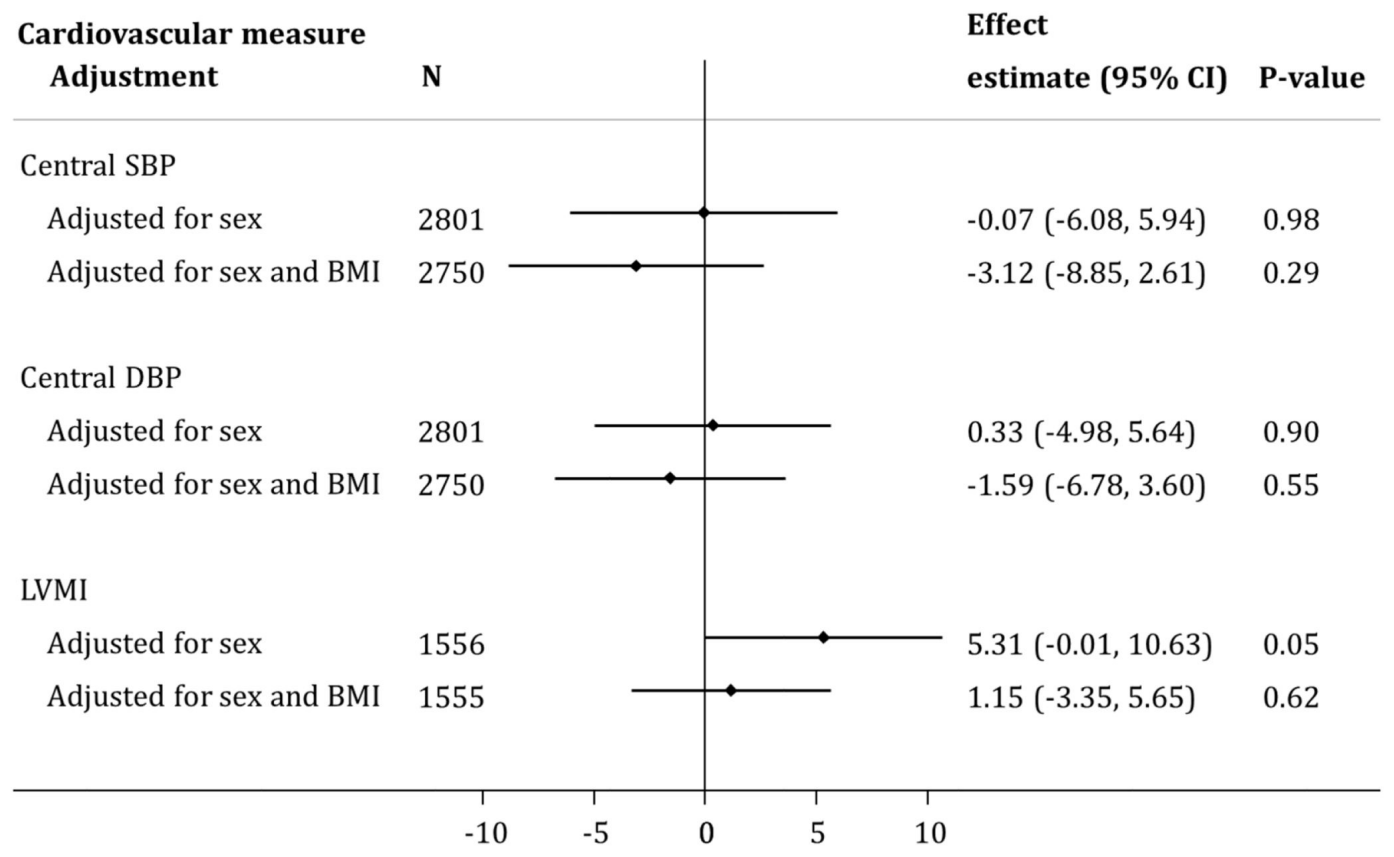
Association of *MC4R* LoF of cAMP accumulation with both central BP and LVMI at age 18 years in a model adjusting only for sex and a model adjusted for sex and BMI at the same age. Estimates represent the change in central DBP (mmHg), central SBP (mmHg) and LVMI (g/m^2.7^) in carriers (i.e., pLoF or cLoF) vs. non-LoF carriers (i.e., individuals with synonymous, common variations or no LoF mutations and individuals with WT-like and GoF mutations) of MC4R LoF mutations, obtained from linear regression (p-values presented are two-sided and not corrected for multiple comparisons). BMI = body mass index; CI = confidence interval; cLoF = complete loss of function; DBP = diastolic blood pressure; GoF = gain of function; LoF = loss of function; LVMI = left ventricular mass index; pLoF = partial loss of function; SBP = systolic blood pressure; WT-like = wild-type like.

Previous studies have reported that carriers of *MC4R* LoF mutations have somewhat lower blood pressure (BP) than equally obese people who are WT at *MC4R^27^*. In this study, there was evidence that *MC4R* LoF mutation carriers had slightly higher systolic blood pressure (SBP) and left ventricular mass index (LVMI) but almost no difference in diastolic blood pressure (DBP) and central BP compared to non-LoF carriers between 8 and 18 years (Extended Data Figure 5 and Extended Data Figure 6). These differences largely attenuated (or, indeed reversed) when adjusting for BMI at the same age. Of note, however, between ages 10 and 12 years, carriers of the *MC4R* LoF mutations had a DBP that was ~3-5mmHg lower after correction for BMI and sex.

### Longitudinal associations with anthropometric traits

Longitudinal analyses were conducted to examine the association between the *MC4R* LoF mutations and the trajectory of BMI, weight and height. Multi-level linear-spline models used to examine longitudinal associations between *MC4R* LoF and anthropometric traits performed well when predicting each trait (Supplementary Tables 8-10).

There was little evidence to suggest that birth weight (mean: 3.44kg) was related to *MC4R* LoF (Supplementary Table 11). The first measure (i.e., intercept of the linear-spline multi-level models) of BMI and height was at 18 months (mean: 16.84kg/m^2^ and 81.90cm, respectively) and there was little evidence that *MC4R* LoF was associated with a difference in either BMI or height at this age in ALSPAC (Supplementary Table 12, Supplementary Table 13).

**Extended Data Figure 7:**
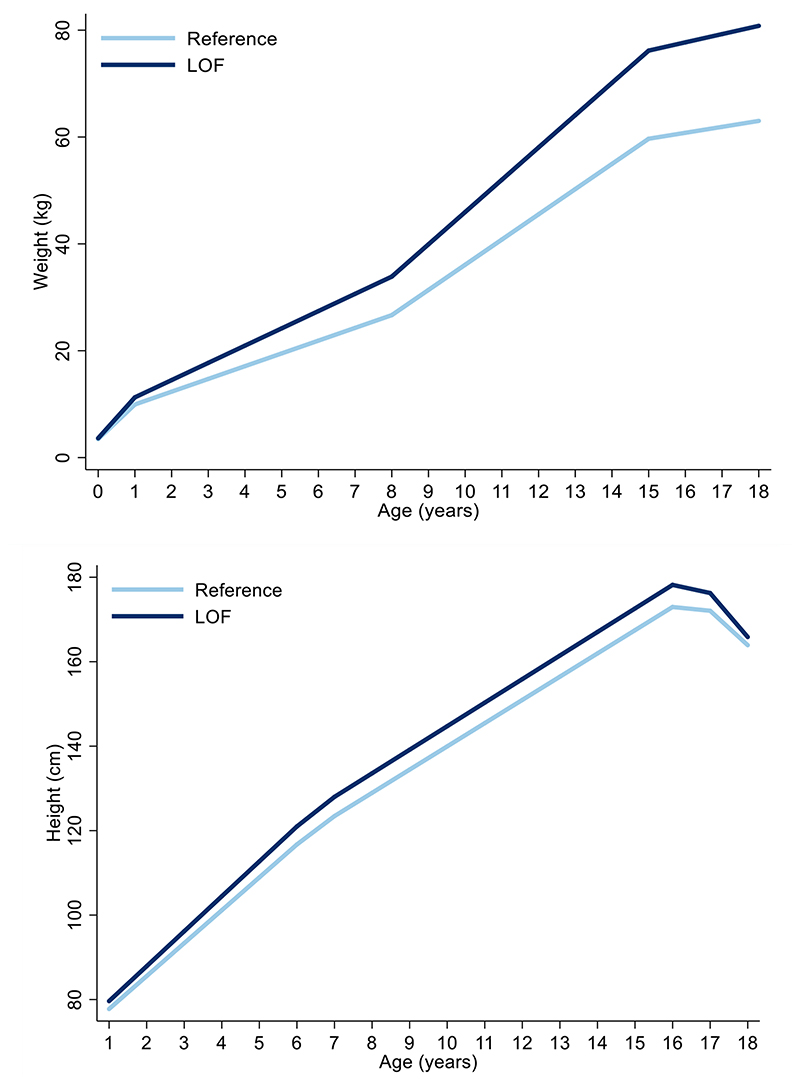
Association between *MC4R* LoF of cAMP accumulation with weight and height trajectories between birth and 18 years using linear spline multi-level models. Values for the reference group (i.e., all individuals with synonymous, common variations or no LoF mutations and individuals with WT-like and GoF mutations) and LoF mutations (i.e., combining pLoF and cLoF mutations) are depicted in light and dark blue, respectively (N=5716). Effect estimates and confidence intervals of these associations are presented in Supplementary Table 11 and Supplementary Table 13 for weight and height, respectively, and were generated using linear spline multi-level models. cLoF = complete loss of function; GoF = gain of function; LoF = loss of function; pLoF = partial loss of function; WT-like = wild-type like.

**Figure 3:**
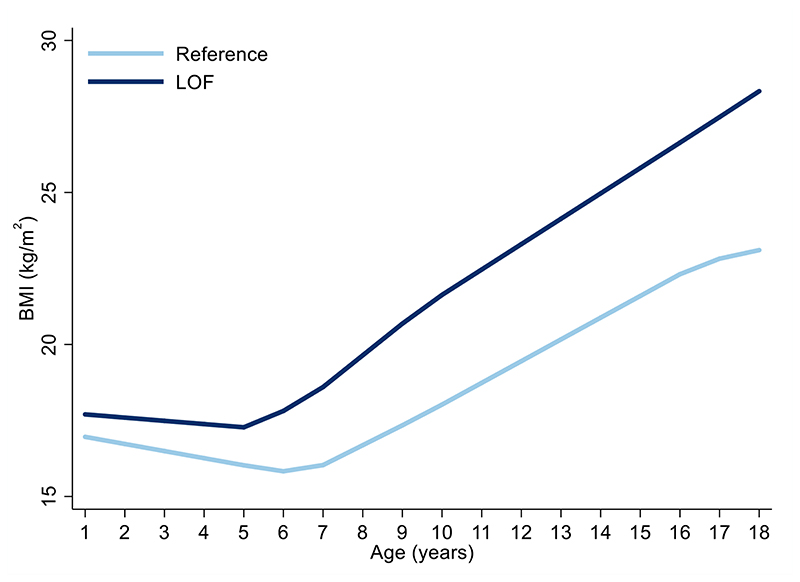
Association between *MC4R* LoF of cAMP accumulation with BMI trajectory between the ages of 18 months and 18 years using linear spline multi-level models Values for the reference group (i.e., all individuals with synonymous, common variations or no LoF mutation and those with WT-like and GoF mutations) and LoF mutations (i.e., combining pLoF and cLoF mutations) are depicted in light and dark blue, respectively (N=5716). Estimates and confidence intervals of these associations are presented in Supplementary Table 12 and were generated using linear spline multi-level models. cLoF = complete loss of function; GoF = gain of function; LoF = loss of function; pLoF = partial loss of function; WT-like = wild-type like.

The effect estimates for the mean difference in BMI change (kg/m^2^ per year in carriers vs. non-carriers of *MC4R* LoF mutations) between 18 months and 18 years were non-zero and consistently positive between the 18 months and 15 years, consistent with the age-specific analyses (Figure 3, Supplementary Table 12). Similarly, the effect estimates for the mean differences in weight change (kg per year) between birth and 18 years were consistently positive, consistent with the age-specific analyses (Extended Data Figure 7, Supplementary Table 11). There was comparatively stronger evidence for a consistently positive effect of *MC4R* LoF on weight change between the ages of 12 months to 8 years (0.84kg/year; 95% CI: 0.40, 1.28; P=1.63x10^-04^) and 8 and 15 years (1.33kg/year; 95% CI: 0.66, 1.99; P=9.62x10^-05^).

There was a modest increase in height with the *MC4R* mutation across childhood and adolescence, with an inverse association in adulthood; however, most confidence intervals for these differences overlapped the null (Extended Data Figure 7, Supplementary Table 13).

### Sensitivity analyses

#### Comparison with β-arrestin-2 coupling

**Extended Data Figure 8:**
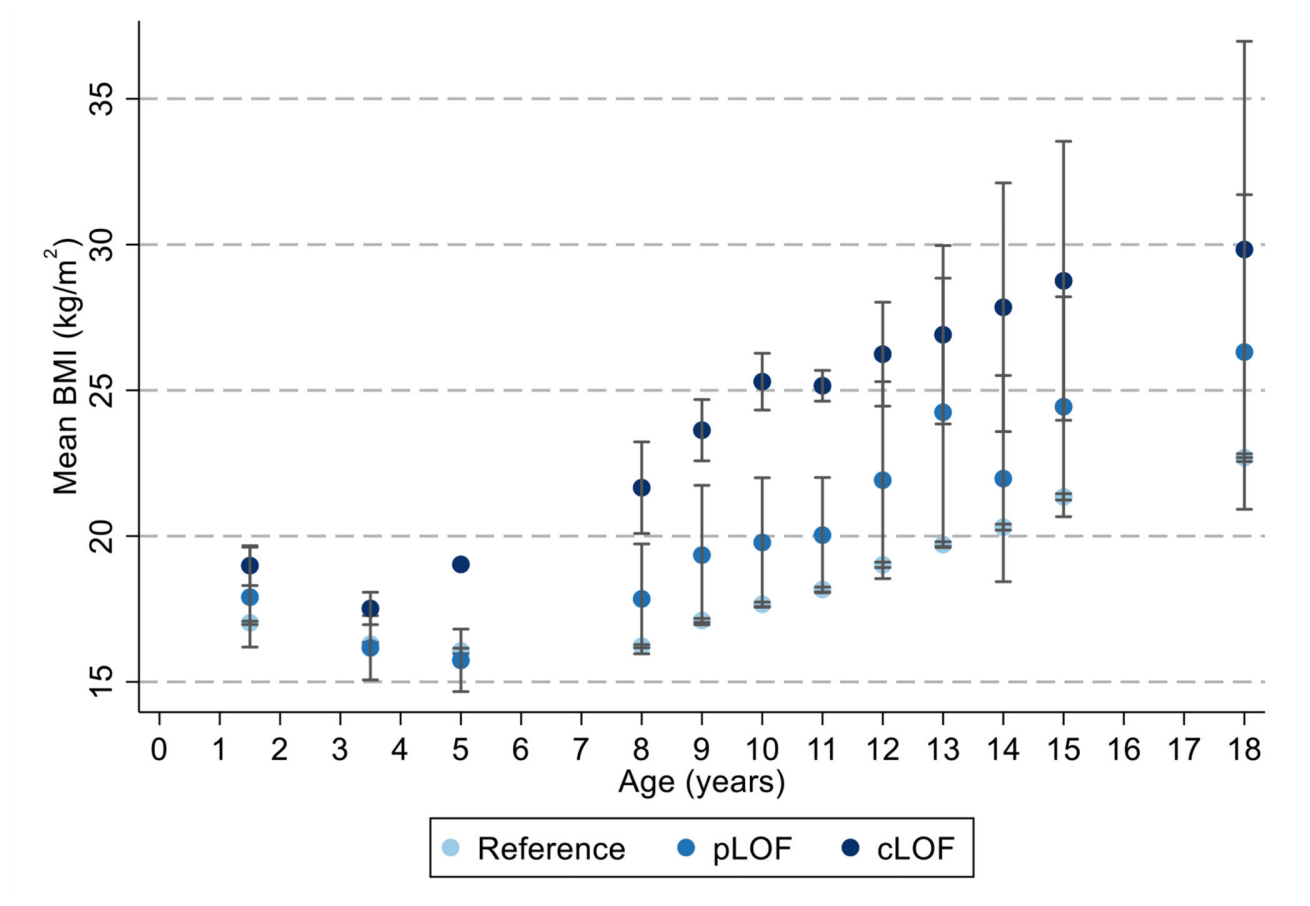
Mean BMI across time with *MC4R* LoF of β-arrestin-2 coupling. Mean BMI +/- 95% CI at different ages (sample sizes across all ages presented in Supplementary Table 14) with MC4R LoF of β-arrestin-2 (carriers of pLoF and cLoF) and the reference group (i.e., non-LoF carriers – combining individuals with synonymous, common variations or no LoF mutations and individuals with WT-like and GoF mutations). Figures only show results where all mutational groups (i.e., WT-like, GoF, pLoF and cLoF mutations) were represented by at least one individual at all time points between birth and 24 years. CI = confidence interval; cLoF = complete loss of function; GoF = gain of function; LoF = loss of function; pLoF = partial loss of function; WT = wild-type.

**Extended Data Figure 9:**
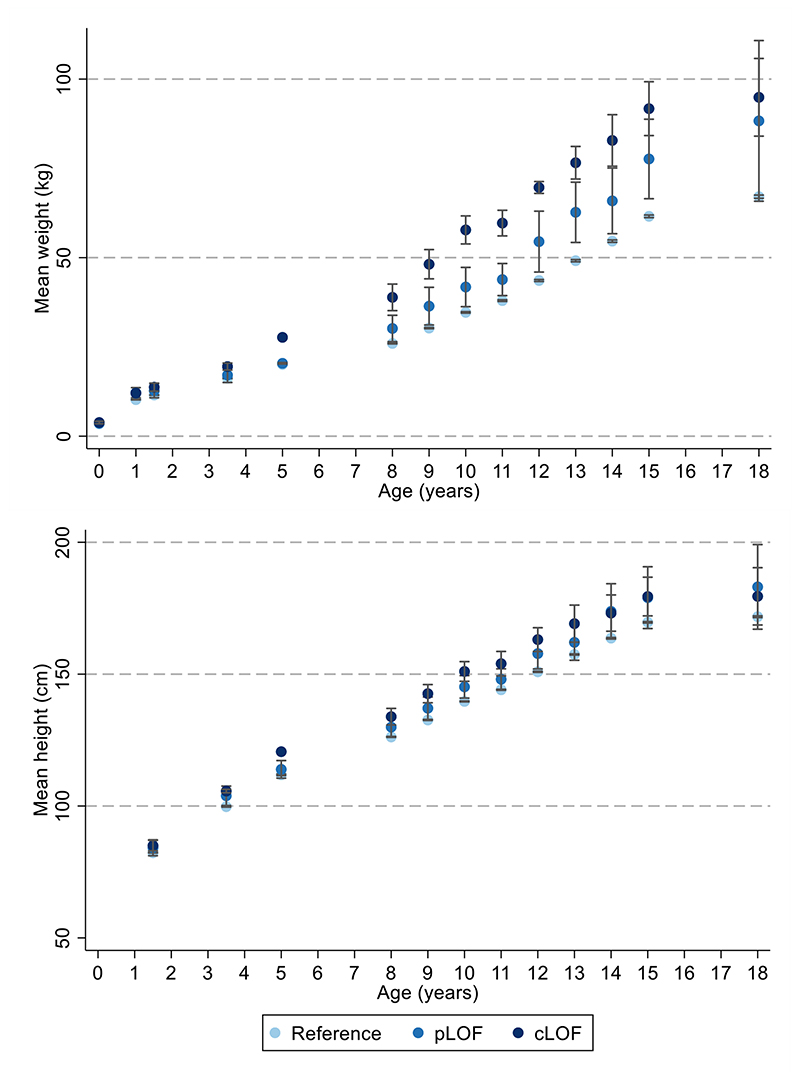
Mean weight and height across time with *MC4R* LoF of β-arrestin-2 coupling. Mean weight and height +/- 95% CI at different ages (sample sizes across all ages presented in Supplementary Table 14) with MC4R LoF of β-arrestin-2 (carriers of pLoF and cLoF) and the reference group (i.e., non-LoF carriers – combining individuals with synonymous, common variations or no LoF mutations and individuals with WT-like and GoF mutations). Figures only show results where all mutational groups (i.e., WT-like, GoF, pLoF and cLoF mutations) were represented by at least one individual at all time points between birth and 24 years. CI = confidence interval; cLoF = complete loss of function; GoF = gain of function; LoF = loss of function; pLoF = partial loss of function; WT = wild-type.

The phenotypic associations of *MC4R* LoF status were largely similar independent of LoF status being defined by β-arrestin-2 coupling or cAMP accumulation assay. There was a consistently positive trend between *MC4R* LoF of β-arrestin-2 coupling BMI from age 3.5 years, and weight and height across the lifecourse (Supplementary Table 14, Extended Data Figure 8 and Extended Data Figure 9), where effect estimates were consistently larger with impairment of β-arrestin-2 coupling from approximately 8 years than cAMP accumulation. Associations between β-arrestin-2 coupling-based classification and fat mass, lean mass and WHR were consistently positive across all time points and had larger (or, with WHR, comparable) effects than those derived with cAMP signalling data (Supplementary Table 15). However, it is worth noting that all confidence intervals of associations of impairment in cAMP accumulation and β-arrestin-2 coupling assays with anthropometric traits overlapped.

#### Comparison between rare and common variation

**Figure 4:**
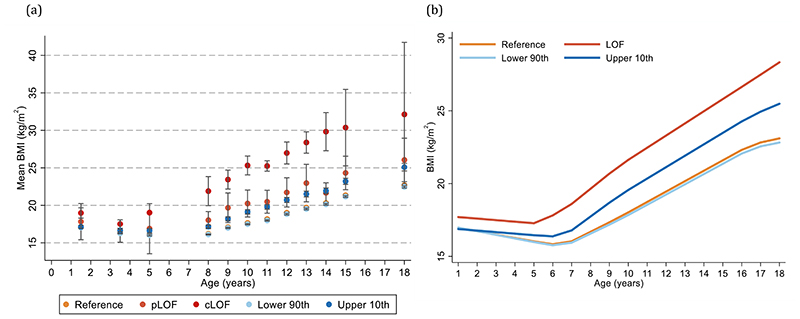
Comparison of the age-specific and longitudinal associations of *MC4R* LoF of cAMP accumulation and a weighted genome-wide polygenic risk score with BMI (a) Mean BMI +/- 95% CI across ages (sample sizes across all ages for MC4R LoF and the weighted gnome-wide polygenic risk score are presented in Supplementary Tables 4 and 16, respectively) and (b) illustration of the BMI trajectory between the ages of 18 months and 18 years using linear spline multi-level models comparing MC4R LoF mutations (N=5716) and weighted genome-wide polygenic risk score (N=5162). Estimates and confidence intervals of the associations with MC4R LoF and the weighted genome-wide polygenic risk score are presented in Supplementary Table 12 and Supplementary Table 18, respectively. Figures only show results where all LoF mutations for MC4R (i.e., pLoF and cLoF mutations) were represented by at least one individual at all time points between birth and 24 years for comparison with the weighted genome-wide polygenic risk score. The reference group included individuals with synonymous, common variations or no LoF mutation and those with WT-like and GoF mutations. BMI = body mass index; CI = confidence interval; cLoF = complete loss of function; GoF = gain of function; LoF = loss of function; pLoF = partial loss of function; WT-like = wild-type-like.

Given the considerable recent interest in the use of genome-wide polygenic risk score (PRS) to predict the development of obesity^28^, we compared the impact of carriage of LoF mutations in *MC4R* with the PRS developed by Khera *et al*.^28^ (comparing upper 10^th^ to lower 90^th^ percentile of the PRS distribution). The magnitude of the effect estimates of *MC4R* LoF on BMI between the ages of 3 and 18 years was approximately double that of obtained by the PRS (Figure 4a, Supplementary Table 16). Similarly, using multi-level linear-spline models, the effect sizes of the change in BMI at 18 months and between 18 months and 18 years were almost always larger with *MC4R* LoF mutation compared to the PRS (Figure 4b, Supplementary Table 17, Supplementary Table 18). Findings from the main analyses of impact of the *MC4R* LoF mutations on BMI were also persistent, albeit slightly attenuated, even when adjusting for the genome-wide PRS (Supplementary Table 19). Unsurprisingly, given the relative rarity of *MC4R* LoF compared to the common SNPs comprising the PRS, the latter explained more of the population variance in BMI than the former (e.g., 0.40% and 10% explained by *MC4R* LoF mutations and the PRS, respectively, in BMI at age 18 years).

## Discussion

By studying a large, representative birth cohort in which anthropometric measures are available throughout childhood, adolescence and early adulthood, we provided estimates of likely frequency for functionally impaired mutations in the *MC4R* gene. In addition to this, we have provided estimates of the phenotypic impact of these mutations during growth and development. We find that mutations at *MC4R* are more frequent and have a consistent and sizeable association with adiposity compared to what has been suggested previously^15^.

To establish the frequency of *MC4R* non-synonymous mutations in a specific population-based study, we developed an approach based on initial pooled high-throughput sequencing. We validated this methodology against WES of individual DNA samples and showed it to have very high sensitivity and specificity. This approach, which has considerable cost advantages, is applicable to the detection of rare, including private, mutations in any gene of interest in large populations.

We estimated a frequency of heterozygous *MC4R* LoF mutations in ALSPAC birth cohort to be 0.30% (with 0.23% carrying pLoF and 0.07% carrying cLoF mutations). Given the well understood demographic characteristics of ALSPAC^23,24^ and notwithstanding ancestry-specific deviations in frequency, it is reasonable to suggest that as many as 1 in every 337 people in the UK could carry a heterozygous LoF mutation in the *MC4R* gene. These estimates are approximately double the previous reports^15, 29–31^ and whilst based on assumptions on the properties of our sample, results here allow a recalibration of prevalence estimates more broadly.

*MC4R* deficiency in mice results in an increase in both fat and lean mass^2^, with early reports suggesting that the same is true in humans^13^. Our results are consistent with these observations, with evidence to suggest a substantial impact of *MC4R* LoF carrier status on BMI, weight, fat mass and lean mass, which was detectable from as early as 5 years. For example, at age 18 years, carriage of an *MC4R* LoF mutation was associated with a 17.76kg greater body weight, a 4.84kg/m^2^ higher BMI and a 14.78kg greater fat mass, with 47.1% of carriers being overweight or obese (≥25kg/m^2^) at that age (compared to 12.6% of non-LoF carriers). Indeed, in our study, 208 participants (3.63%) of the 5724 individuals who were sequenced were obese (BMI >30kg/m^2^) at age 18 years and, of these, 0.96% had LoF mutations in *MC4R* (compared to the 0.27% individuals who carried LoF mutations in *MC4R* out of 5516 individuals who were not obese). *MC4R* deficiency has also been reported to be associated with an increase in linear growth velocity attributable to hyperinsulinemia and the absence of the suppression of growth hormone levels that is usually seen in other forms of obesity^32^. Consistent with this, we observed a trend towards increased height with *MC4R* LoF during longitudinal follow-up.

In a recent study of UK Biobank participants, Turcot *et al*. reported a somewhat smaller impact of carriage of a heterozygous nonsense mutation in *MC4R* on body weight and the prevalence of obesity, where the majority of carriers were not obese^20^. Whilst we similarly found that not all carriers were, indeed, obese (in fact, the distributions of carriers and non-carriers of *MC4R* LoF mutations overlapped in most cases), the impact of carriage of such LoF mutations was, as described, more substantial. However, these previous findings by Turcot *et al*. should be viewed in the light of the known selection bias in UK Biobank^33^, whose participants are on average lighter and healthier than unselected members of the UK population and the fact that the particular subset of UK Biobank participants analysed in this study contained a sub-population that disproportionately represented heavy smokers.

In the current analyses, carriers of mutations that were functionally WT-like were practically indistinguishable from other non-LoF carriers or carriers of GoF mutations in their anthropometric characteristics. This emphasises the importance of knowing the functional impact of any non-synonymous mutation found during diagnostic testing in obesity. Indeed, databases collating information on the likely pathogenicity of all known mutations (https://www.mc4r.org.uk/) are very helpful to clinicians in this regard but, until every possible mutation has been systematically generated and characterised as has been undertaken with *PPARG* ^34^, for example, such databases will remain incomplete. The mutations found in the present study had a largely similar impact on β-arrestin-2 coupling and cAMP accumulation (and, thus, had a largely similar impact on anthropometric traits) and do not, therefore, contribute to addressing questions around the relevance of biased signalling.

We previously reported that obese patients with *MC4R* LoF mutations have lower BP compared to similarly obese non-LoF carriers^27^. In this study, while there was a trend for an inverse association between *MC4R* LoF and BMI-adjusted measurements of both arterial and central cardiovascular health, there was no clear indication of lower BP in the *MC4R* carriers across the lifecourse. This finding, albeit restricted to a limited age range and sample size, is consistent with previous observations regarding the cardiovascular effects of *MC4R* functional impairment^27^.

Genome-wide PRSs associated with BMI have recently received considerable attention as possible predictors of phenotypes such as obesity^28^. In that context, it is notable that the impact of carrying a functionally impaired *MC4R* locus on BMI was approximately double that of the common PRS used previously (comparing the lower 90^th^ and upper 10^th^ percentiles of the continuous PRS distribution). Indeed, this was seen to be an effect which was persistent after having adjusted for PRS. This observation is not incompatible with the possibility of a buffer or enhancer-effect being present as a result of the individual-level combination of rare genetic changes and PRS value^35^. However, results here do suggest that the rare changes at *MC4R* are likely to have a larger impact than more subtle and continuous on-average differences delivered by theoretically additive PRS contributions at an individual level - a contrast to the nature of effect when considering total population variance explained.

A particular advantage of ALSPAC is the availability of robust longitudinal phenotyping data throughout childhood, adolescence, and adulthood. Childhood obesity is strongly associated with adverse cardiometabolic outcomes in later life^36^. However, it appears that the long-term adverse health consequences of childhood obesity are driven by the tendency of the obese phenotype to persist into adult life^37,38^. While it is conceivable that rescue of the phenotype at this stage of development would reduce cardio-metabolic risk (this also suggested in our analyses adjusting for BMI), we know from a longitudinal assessment of adult *MC4R* mutation carriers, that penetrance of the phenotype increases with age^19^. It therefore seems likely that the obese phenotype of *MC4R* LoF mutation carriers in ALSPAC cohort will persist or may even worsen with age.

Main limitations of our study broadly include the sample size and representative value of the ALSPAC study with respect to the wider population. Firstly, there was a relatively small number of individuals carrying *MC4R* LoF mutations identified in the ALSPAC participants sequenced. Despite this, we were able to identify, validate and functionally characterise *MC4R* LoF mutations in addition to assessing their downstream impact on adiposity-related phenotypes within the current sample. The estimates presented should be taken in the context of their precision and overall pattern in this longitudinal data, which we have displayed transparently. Of course, we are likely to be underpowered to detect *MC4R* LoF mutations occurring at a lower frequency in this population, so further analyses such as those presented here conducted in larger populations are warranted.

Secondly, it is important to acknowledge the limited representation that ALSPAC offers to other populations, even those that are predominantly of European descent. There was evidence for differences in some lifestyle, socioeconomic and anthropometric traits between those sequenced and not sequenced and, whilst most differences were indeed negligible in real terms, these differences suggested an overrepresentation of healthier individuals of higher socioeconomic position. This may imply that the frequencies and associations of *MC4R* LoF mutation presented here may not be totally representative of the wider UK and beyond; however, it is difficult to ascertain whether results presented here are over- or under-estimates of these characteristics. No single birth cohort, no matter how comprehensively collected, can be assumed to be representative of a complete target sample. Furthermore, given the initial sampling frame for ALSPAC, which captured>80% of all pregnancies in the Greater Bristol region of the UK in the early 1990s, the study is likely more representative of an unselected population of the wider UK than other sampling initiatives based on adult volunteers conducted within this field. Whilst greater precision around frequency and associational estimates of *MC4R* LoF mutations described in this study would certainly be afforded by a larger sample, the accuracy of derived estimates would similarly be subject to the representation of such samples.

Our study suggests that *MC4R* LoF mutations contribute substantially to adiposity traits, with effects starting in early childhood and persisting into adult life. Estimates here are complementary to existing studies, though naturally vary given the sampling frame and data type reported here^26^. Despite this, our work suggests that heterozygous mutations that substantially impair the function of the *MC4R* gene may very well be found in several millions of people worldwide and will tend to increase the body weight and adiposity from an early age and persist across the lifecourse. Given the established association between *MC4R* LoF mutations and the complications of obesity such as type 2 diabetes and coronary artery disease, this is of substantial clinical importance to the long-term health of individual carriers who will, on average, likely enter adult life carrying ~15kg of extra fat mass. With a prevalence of ~1/340, Melanocortin 4 receptor deficiency can no longer be considered a “rare disease”, the definition of which, in the UK, is a prevalence of <1/2000 and, in the US, is <200,000 affected patients nationally. If the prevalence in the USA reflects that found in the UK, we would predict that there are around a million Americans whose weight is substantially increased by the carriage of an *MC4R* mutation. Efforts to reduce obesity and maintain a healthy weight in carriers of *MC4R* LoF mutations, through diet and physical activity will likely need to begin early in life and be targeted in nature, to have an optimal chance of reducing the risks of developing obesity later in life. Pharmacological enhancement of residual intact melanocortin signalling could provide a clinically useful complement to such measures in these patients. The likely size of the population affected should help to stimulate investment in such therapeutic approaches.

## Methods

### Study sample and measures

The Avon Longitudinal Study of Parents and Children (ALSPAC) is a large geographically-homogeneous prospective birth cohort from the southwest of England established to investigate environmental and genetic characteristics that influence health, development and growth of children and their parents^23,24,39^. Full details of the cohort and study design have been described previously and are available at http://www.alspac.bris.ac.uk. Please note that the study website contains details of all the data that is available through a fully searchable data dictionary and variable search tool (http://www.bristol.ac.uk/alspac/researchers/our-data/).

Briefly, 14541 pregnant women residing in the former county of Avon with an estimated delivery date of between the 1^st^ of April 1991 and the 31^st^ of December 1992 (inclusive) were enrolled to the study. Out of those initially enrolled, 13988 children who were alive at 1 year of age and have been followed up to date with measures obtained through regular questionnaires and clinical visits, providing information on a range of behavioural, lifestyle and biological data. When the oldest children were approximately 7 years of age, an attempt was made to bolster the initial sample with eligible cases who had failed to join the study originally. As a result, when considering variables collected from the age of seven onwards (and potentially abstracted from obstetric notes), there are data available for more than the 14541 pregnancies mentioned above. The number of new pregnancies not in the initial sample (known as Phase I enrolment) that are currently represented on the built files and reflecting enrolment status at the age of 24 is 913 (456, 262 and 195 recruited during Phases II, III and IV respectively), resulting in an additional 913 children being enrolled. The phases of enrolment are described in more detail in the cohort profile paper^23^.

The total sample size for analyses using any data collected after the age of seven is therefore 15454 pregnancies, resulting in 15589 foetuses. Of these 14901 were alive at 1 year of age. A 10% sample of the ALSPAC cohort, known as the Children in Focus (CiF) group, attended clinics at the University of Bristol at various time intervals between 4 to 61 months of age. The CiF group were chosen at random from the last 6 months of ALSPAC births (1432 families attended at least one clinic). Those excluded were mothers who had moved out of the area or were lost to follow-up, and those partaking in another study of infant development in Avon.

Ethical approval for the study was obtained from the ALSPAC Ethics and Law Committee and the Local Research Ethics Committees. Consent for biological samples has been collected in accordance with the Human Tissue Act (2004) and Informed consent for the use of data collected via questionnaires and clinics was obtained from participants following the recommendations of the ALSPAC Ethics and Law Committee at the time. Written informed consent was obtained from mothers at recruitment, from the main carers (usually the mothers) for assessments on the children from ages 7 to 16 years and, from age 16 years onwards, the children gave written informed consent at all assessments.

Academic attainment was derived by questionnaire asking whether the participant was still in full-time education (with possible answers of “yes” and “no”), when the participant was aged 18 years. Participant sex was measured from the birth notification as part of the cohort profile. Participant ethnicity was defined as either “White” or “Non-white” based on a questionnaire issued at approximately 32 weeks gestation completed by the participant’s mother. Participant ancestry was confirmed using multi-dimensional scaling (MDS) on 1000 genomes imputed data available in the ALSPAC sample. Household income was defined as the family income (in pounds) per week when the participant was 33 months old (defined as <£100, £100-199, £200-299, £300-399 or >£400). Age of mother at the birth of her first child was taken from a questionnaire administered during the 18-20 weeks gestational period of the ALSPAC participant. Maternal pre-pregnancy body mass index (BMI) was derived from weight (kg) and height (cm) measures obtained from a questionnaire administered during her pregnancy with the ALSPAC participant and calculated as weight divided by the square of height (kg/m^2^). Maternal weight gain was taken from obstetric records, calculated as the absolute weight gain from the last minus the first weight measurement (kg).

Highest household social class was a derived variable reflecting the highest social class based on occupation held by the participant’s mother or mother’s partner at 18 weeks gestation (with levels including “I – Professional”, “II – Managerial and technical”, “IIINM – Skilled non-manual”, “IIIM – Skilled manual”, “IV – Partly skilled” and “V – Unskilled”). Maternal and paternal education were derived from a questionnaire administered to the participant’s mother at 32 weeks gestation asking whether she and her partner had various qualifications, combined into a single variable reflecting her and her partner’s highest educational qualification (with levels including “CSE/none”, “Vocational”, “O-level”, “A-level” and “Degree”, where “CSE” is a Certificate of Secondary Education). Parity was defined as the number of previous pregnancies the participant’s mother had that resulted in either a live- or still-birth, obtained from a questionnaire administered at 18-20 weeks gestation.

Length and weight of each participant were measured at birth and at 4, 8, 12 and 18 months. Height (to the nearest millimetre) and weight (to the nearest 50g) were measured from 25 months to 24 years. For weight, the participant was encouraged to pass urine and undress to their underclothes. For height, children were positioned with their feet flat and heels together, standing straight so that their heels and shoulders came into contact with the vertical backboard. Equipment used (e.g., Harpenden Neonatometer or Stadiometer, Kiddimetre and Leicester measure for height and the Fereday 100kg combined scale, Soenhle scale, Seca scale and Tanita Body Fat Analyser for weight) for each measurement were comparable. In addition to the height and weight measures obtained at ALSPAC clinics, weight and height measures derived from other sources (specifically, mother-completed questionnaires and health visitor records) between the ages of four months and when participants were 10 years were used to supplement available clinic values^40^. BMI at all ages was calculated as weight or length (kg) divided by height (m) squared.

Both waist and hip circumferences were measured when the participants were a mean age of 8, 10, 12 and 24 years. Waist circumference was measured to the nearest millimetre at the minimum circumference of the abdomen between the iliac crests and the lowest ribs. Hip circumference was measured to the nearest millimetre at the point of maximum circumference around the participant’s hips. Waist-hip ratio (WHR) was calculated as the ratio of these two measurements.

Fat and lean masses (kg) were measured when participants were a mean age of 10, 12, 14, 15, 18 and 24 years using the Lunar prodigy narrow fan beam densitometer dual energy x-ray absorptiometry (DXA) scanner. The participant was asked to lie on the machine (in light clothing without any metal fastenings) and encouraged to keep as still as possible whilst the arm of the machine moved over, and two sources of X-ray scanned the participant.

Arterial blood pressure (BP) was measured when participants were a mean age of 3, 4, 5, 8, 10, 12, 13, 14, 15, 18 and 24 years old, with the appropriately sized cuff. Equipment used included Dinamap vital signs monitors (models 9300, 9301 and 8100) and Omron oscillometric devices (models MI-5, 705 IT, IntelliSense M6 and BP Cuff), which were comparable. Additionally, when participants were a mean age of 18 and 24 years, measures of cardiac structure and function were obtained. Of these measures, we used information about central BP and left ventricular mass index scaled by height to the power of 2.7 (LVMI, g/m^2.7^), as proxies for cardiovascular health^41^. Central BP was measured using radial artery tonometry with a SphygmoCor Px Pulse Wave Analysis System (Atcor Medical) at both age 18 and 24 years. Echocardiography was performed using a HDI 5000 ultrasound machine (Phillips) and P4-2 Phased Array ultrasound transducer (at age 18 years) and a Philips EPIQ 7G Ultrasound System (at age 24 years) using a standard examination protocol and left ventricular mass was estimated according to American Society of Echocardiography guidelines^42^.

Full details of all measures used in this study are available on the online dictionary: http://www.bristol.ac.uk/alspac/researchers/our-data/.

### Detection of *MC4R* mutations by pooled sequencing

**Extended Data Figure 10:**
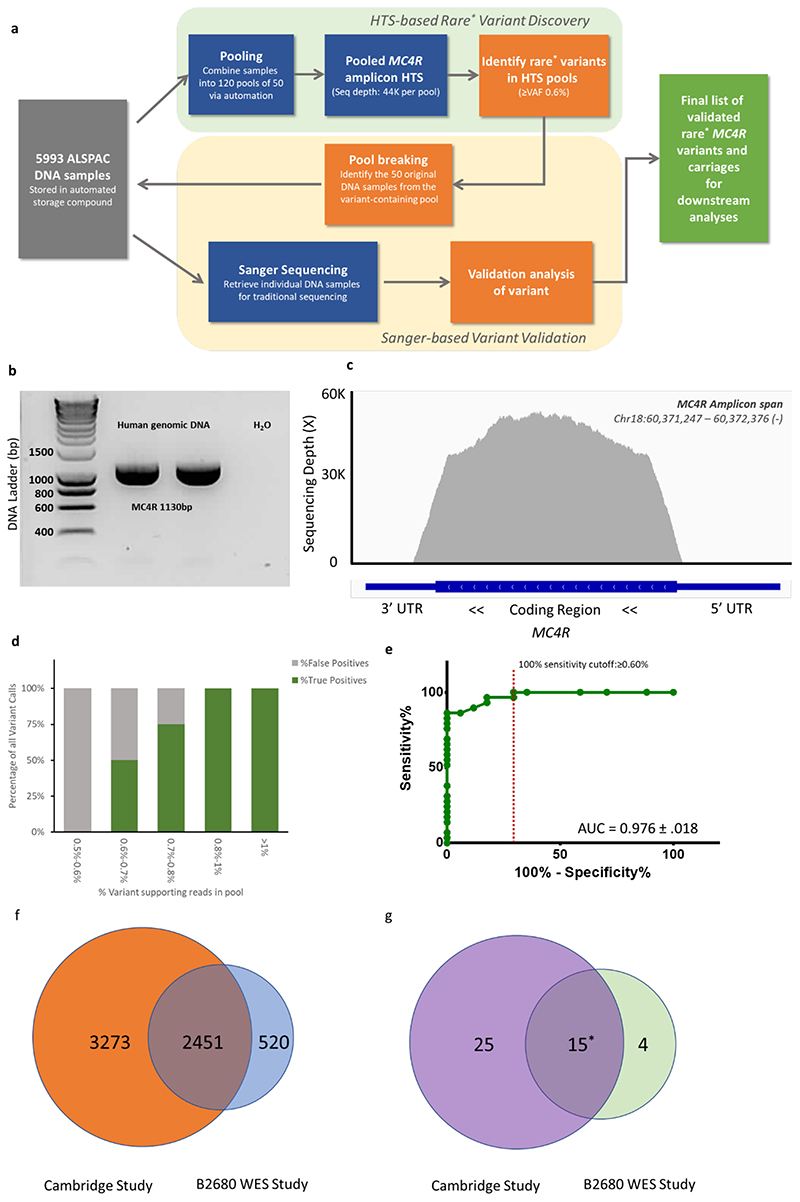
Identification of rare *MC4R* variants by pooled sequencing. (a) Pooled MC4R sequencing workflow for DNA samples from the ALSPAC cohort; (b) Agarose gel showing a 1130bp PCR product using MC4R exon primers (Supplementary Table 1), PCR products from 2 independent DNA samples are shown; (c) A plot showing the sequencing coverage of MC4R coding region from a representative pool. The average perbase sequencing depth for all pools was 43,654 ± 356-fold; (d) The percentage of true positive and false positive calls binned by variant allele frequency (VAF) detected in pools; (e) Receiver operating curve analysis of VAF and call accuracy; (f) Comparison of participants included in the current study and another whole-exome sequencing (WES) study (with ALSPAC project number: B2680); (g) Comparison of non-synonymous variant carriages between this study and WES study. Of 40 mutational carriages found in Cambridge, 15 were found in both studies. 25 carriers found in the Cambridge study were not part of the WES study and 4 carriers found only in the WES study were not part of the Cambridge study. *rare = minor allele frequency < 0.01%, both p.V103I and p.I251L were above 0.01% and excluded in the analyses. ALSPAC = Avon Longitudinal Study of Parents and Children; AUC = area under the curve; HTS = high-throughput sequencing; WES = whole-exome sequencing.

The pooled *MC4R* sequencing workflow used in the study is shown in Extended Data Figure 10. The workflow was broadly divided into the ‘Discovery’ phase and the ‘Validation’ phase. In the ‘Discovery’ phase’, a small aliquot of the original DNA sample was combined with 49 other samples into a DNA pool, and this was followed by high-throughput sequencing (HTS) of the pool to identify variation in the *MC4R* gene. Next, in the second ‘Validation’ phase, for each pool containing one or more variants of interest (in this case rare variants with MAF <0.01%), we went back to all of the 50 original DNA samples and re-sequenced these variants using the traditional Sanger method. The main objectives for this phase were to: 1) orthogonally validate the variant discovery from HTS; 2) identify the variant carriage; and 3) establish the zygosity of the carriage.

#### Pooled HTS of MC4R

20ng of 5993 DNA samples from ALSPAC were randomly combined into pools of 50 at the Medical Research Council Biorepository Unit. 10ng of pooled DNA was used for *MC4R* exon PCR with Q5 Hot Start High-Fidelity DNA Polymerase (NEB, Ipswitch MA, USA) and *MC4R* exon primers -27 bp upstream and +104 bp downstream of the protein coding region (Extended Data Figure 10, Supplementary Table 20). The PCR product was purified using Agencourt Ampure XP beads (Beckman Coulter, Brea CA, USA), and quantified using the QuantiFlour dsDNA system (Promega, Wadison, WI, USA) and Tecan Infinite M1000 Pro plate reader (Männedorf, Switzerland). Sequencing libraries were constructed from 1ng of purified PCR product using the Nextera XT Library Preperation Kit with Nextera XT Index V2 barcodes (Illumina, San Diego, CA, USA) according to manufacturer’s instruction. The final libraries were purified using Agencourt Ampure Xp beads. Purified libraries were quantified by real-time quantitative PCR using the Kapa Library quantification kit (Roche, Basel, Switzerland) on a Quantstudio 7 Flex Real Time PCR instrument (ThermoFisher scientific, Waltham, MA, USA). Finally, the libraries were combined at 10nM for sequencing both ends for 150bp (PE150) on the Illumina HiSeq 4000 instrument at the CRUK Cambridge Institute Genomics Core. We achieved an even coverage throughout the protein coding region of *MC4R*, with a mean sequencing depth at 43,654 ± 356-fold per pool (Extended Data Figure 10).

#### Sequencing bioinformatics

Sequence reads were mapped using BWA MEM algorithm (version 0.7.12) onto the Human GRCh38 (hg38) genome. PCR duplicates were removed using Picard version 1.127 followed by indel realignment and base quality score recalibration using GATK version 3.8 according to GATK Best Practices. The variant calls were generated by Varscan version 2.4.2 mpileup2snp and mpileup2indel function with the following criteria: variant allele frequency (VAF) ≥0.05%, coverage ≥100, p-value <0.05 and strand filter set to ON. To maximise variant detection sensitivity, we started with an initial VAF cut-off at 0.5%, which was lower than the theoretically value of 1% in order to allow for technical errors and experimental bias, with an expectation of detecting false positives (FPs). The cut-off was readjusted using validation results from Sanger sequencing (see below).

#### Variant validation and rare variant carrier identification

Original DNA samples from all rare variant containing pools (except p.V103I and p.I251L) were retrieved for variant validation using traditional Sanger sequencing. The *MC4R* coding region was amplified, using GoTaq Green (Promega) Master Mix with 10ng DNA per 10μl PCR reaction and *MC4R* exon primers used in NGS (Supplementary Table 20). *MC4R* PCR cycling conditions were as follows: one cycle of Hot Start at 95°C for five minutes, then 35 cycles of the following: denaturation at 95°C for 30 seconds, annealing at 60°C for 30 seconds, extension at 72°C for 2 minutes. Then, one cycle of final extension at 72°C for five minutes.

Unincorporated primers and dNTPs were removed from the PCR reactions by digesting with Exonuclease I (Exo) (NEB) and Shrimp Alkaline Phosphatase (SAP) (NEB) as follows: 20 units of Exonuclease I and 1 unit of Shrimp Alkaline Phosphatase were added directly to the 10 μl PCR reaction; the EXO/SAP reaction was then incubated at 37°C for 20 minutes and then the enzymes were deactivated by incubating at 80°C for 15 minutes. This EXO/SAP PCR reaction was then used as the template for the Sanger Sequencing reaction.

Sanger sequencing reactions were set up using BigDye Terminator v3.1 Cycle Sequencing Kit (Thermal Fisher) in a 10μl reaction using 0.5μl of BigDye Terminator v3.1, 2μl 5x Sequencing buffer, 0.5μM sequencing primer and 1μl of the EXO/SAP PCR product which was made up to 10μl using Nuclease free water. The Sanger sequencing cycling conditions were as follows: denaturation at 95°C for 10 seconds, annealing at 50°C for five seconds, extension at 60°C for four minutes. This program was continued for 24 cycles in total.

Sanger sequencing reactions had unincorporated dye and primers and dNTPs removed using AxyPrep MAG PCR Clean-Up Kit (Axygen) according to manufacturer’s instructions. Purified sequencing products were resuspended in 30μl nuclease free water. Sanger sequencing reactions were analysed on a 3730 DNA Analyzer (Thermal Fisher). Sanger sequencing data files were analysed using Sequencher version 4.8 Build 3767 (Gene Codes Corporation).

#### Specificity and sensitivity of pooled sequencing

Excluding p.V103I and p.251L, the initial screen using a VAF 0.5% cut-off resulted in a total of 38 rare, non-synonymous *MC4R* variants with an estimated carriage of 57 individuals. Of these, 40 individuals carrying 27 unique variants were confirmed as true positives (TP) by Sanger sequencing (Extended Data Figure 10). The mean (± standard deviation, SD) VAF detected for TP was 1.18 (± 0.39%). We found a strong relationship between VAF and specificity, where all variants called at VAF<0.60% were FP (Extended Data Figure 10), this indicates the likelihood of missing any true potential variants at VAF of <0.5% was extremely low. We also performed receiver operating curve (ROC) analysis using GraphPad Prism version 6 and showed that VAF was a strong predictor for variant detection (AUC=0.976, Extended Data Figure 10). Using findings from ROC, we adopted a final VAF cut-off at ≥0.60% and improved the specificity to 88.89%, whilst retaining all the 40 TP calls for downstream analysis.

To establish method sensitivity, we compared our *MC4R* variant call set with another ALSPAC whole-exome sequencing (WES) study of 2971 individuals. The *MC4R* locus in this study was sequenced at a depth of 28.15X. Within the overlap of 2451 (out of 5724) unique individuals sequenced in both studies (Extended Data Figure 10), we found that the TP variant call sets were 100% concordant (Extended Data Figure 10). This implies the sensitivity from our novel pooled sequencing method was on par with standard WES.

### Functional characterisation of *MC4R* mutations in vitro

#### cAMP accumulation assay

CV-1 in Origin with SV40 genes 7 (COS-7) cells were maintained in a growth medium containing low glucose Dulbecco’s modified eagle medium (Invitrogen, Carlsbad, CA, USA), 10% fetal bovine serum (Invitrogen), 1% Glutamax (Invitrogen), 100 U/ml penicillin and 100 mg/ml streptomycin (Sigma-Aldrich, IL, USA). COS-7 cells were kept at 37°C humidified air with 5% CO_2_.

Site-directed mutagenesis was performed on WT Human N-FLAG-MC4R PCDNA3.1(+) using Agilent QuikChange Lightning kit (Santa Clara, CA, USA) to generate all 7 previously uncharacterised *MC4R* variants for cAMP activity measurement.

30ng of plasmid carrying *MC4R* WT and variants were transfected into COS-7 cells using (Lipofectamine 2000, Invitrogen) for 24 hours. [Nle^4^,D-Phe^7^]-α-melanocyte-stimulating hormone (NDP-αMSH, Bachem, Bubendorf Switzerland), dissolved in 0.1% bovine serum albumin (BSA) and 1mM acetic acid at a stock concentration of 5mM, was added to cells at increasing final concentrations of 10^-12^ to 10^-6^M in the growth medium for 2 hours, before intracellular cAMP concentration measurement using a luminescence based HitHunter cAMP Assay for small molecules (Cat# DiscoverX 90-0075SM25 Eurofins DiscoverX, Fremont, CA, USA) and a Tecan Spark 10M microplate reader. The baseline and maximal luminescence signal was normalised to *MC4R* WT and a 4-point sigmoidal dose-response curve was fitted to normalised values from all replicates to determine the E_max_ and logEC_50_ using Graphpad Prism version 7. Differences in estimated logEC_50_ and E_max_ were determined using an extra sum-of-squares F-test. Due to the lack of response, we did not perform a curve fit for complete LoF (cLoF) variants and only determined the relative E_max_ based on cAMP level measured at 10^-6^M NDP-αMSH and the difference was measured using a two-sided student t-test.

#### B-arrestin-2 coupling assay

To examine the interactions between MC4R and β-arrestin-2, we used the NanoBiT protein/protein interaction assay (Promega). *MC4R* WT and variants were cloned into the pBiT1.1-C(TK/LgBiT) vector. 50ng of the *MC4R*-LgBiT and *ARRB2*-SmBiT were co-transfected into HEK293 cells as described by Lotta., *et al*. 2019^26^. Human embryonic kidney 293 (HEK-293) cells were maintained in high glucose Dulbecco’s modified eagle medium (Invitrogen), 10% fetal bovine serum (Invitrogen), 1% Glutamax (Invitrogen), 100 U/ml penicillin and 100 mg/ml streptomycin (Sigma-Aldrich, St Louis, MO, USA). HEK-293 cells were kept at 37°C humidified air with 5% CO_2_. 24 hours after transfection, culture medium was replaced with Opti-MEM I medium (Invitrogen) 30 minutes before luciferase activity was measured by the Tecan Spark 10M microplate reader set at 37°C and 5% CO_2_. After 2.5 minutes, 20μl of Nano-Glo Live Cell Assay System (Promega) was added and luciferase activity was measured for 10 minutes to generate the baseline signal. Cells were then stimulated with NDP-αMSH at 10^-12^ to 10^-6^M and luciferase activity was monitored for another 30 minutes. The area under the curve (AUC) above the baseline was then used to determine the coupling between MC4R and β-arrestin-2. For each individual experimental replicate, the AUC values were normalised to % maximum AUC of *MC4R* WT from the same experiment and a 3-point sigmoidal dose-response curve was fitted to determine E_max_ and logEC_50_. The average E_max_ and logEC_50_ values were used for LoF determination. The logEC_50_ was not used for cLoF mutants that exhibited no response. All calculations were performed with GraphPad Prism 6. Differences in the mean logEC_50_ and E_max_ were determined using a two-sided student’s t-test.

#### Identification of rare variant carriers

Once we completed the functional characterisation of *MC4R* mutations, we unencrypted sequenced pools to identify specific individuals carrying these mutations. This allowed the phenotypic characterisation of such functional impairment of *MC4R*. Of the 5993 individuals sequenced, five individuals had missing identifier information for linkage with the wider ALSPAC data set and 214 individuals were duplicated; therefore, these exclusions left 5774 participants in the sequenced set (note that none of the excluded individuals had an *MC4R* LoF mutation). After merging in all required clinic and questionnaire data from the ALSPAC cohort, there were related individuals (i.e., siblings) within the total sample. For appropriate comparisons between those included within and excluded from the sequenced set, all related individuals were excluded. Specifically, there were one set of quadruplets (none of which were in the sequenced set), four sets of triplets (one full set of which was in the sequenced set) and 255 sets of twins (48 complete sets of which were in the sequenced set). In addition to these 48 pairs of twins in the sequenced set, there were 35 sets of twins where one twin was in the sequenced set and the other twin was not in the sequenced set. To avoid removing as many individuals from the sequenced set as possible, the twin not in the sequenced set was removed in these instances (n=35).

A total of 216 individuals were excluded from the total sample to remove siblings (note that, at this point, none of these were from the sequenced set), which included 35 individuals (one of a pair of twins not in the sequenced set), three individuals from a quadruplet, two individuals each from three triplets (n=6), 172 individuals from twin sets. Then, siblings in the sequenced set were removed, which included the two individuals from the one triplet set and one individual from each of the 48 twins (n=50). After all exclusions, there were 5724 left in the sequenced set for all analyses.

To identify and estimate the prevalence of carriers of loss of function (LoF) mutations, tabulations were used, separated by gain of function (GoF), wild-type-like (WT-like), partial LoF (pLof) and cLoF mutations, in the sequenced set of ALSPAC participants.

### Statistical analyses

For this paper, we focused on *MC4R* LoF of cAMP production as our main analysis, with comparison to impairment of β-arrestin-2 coupling and to a genome-wide polygenic risk score (PRS) comprising over 2 million common genetic variants as sensitivity analyses. There was little evidence for a difference in the phenotypic effect of *MC4R* LoF mutations across ethnicities (all estimates and confidence intervals overlapped) and no evidence of overt relatedness (maximum values were 10 times less than first cousins) across mutation carriers. All analyses were conducted using Stata (versions 15 and 16) and MLwiN version 3.04 called from Stata using the *runmlwin* command^43^.

#### Representation

To explore how representative the participants of the sequenced set were of the wider ALSPAC cohort, measures of education, socioeconomic status and parental factors were compared between individuals within the sequenced set and those not in the sequenced set. These variables were selected based on those used in previous papers comparing the ALSPAC cohort with statistics from a national sample of the United Kingdom^23,24^. These measures included academic attainment, sex, ethnicity, household income, maternal age at birth of first child, maternal pre-pregnancy BMI, maternal weight gain during pregnancy, highest household social class, parental education, and parity (see above for details on how these were measured).

Means and SDs of all continuous variables and percentages of binary or categorical variables were calculated and t-tests were used to test whether summary statistics were different between participants included in the sequenced set and those in the wider ALSPAC cohort.

#### Age-specific associations with anthropometric traits

Age-specific analyses were conducted using linear regression across all available measures of BMI, height, weight, WHR, fat mass and lean mass between birth and 24 years. All individuals with data on the *MC4R* mutations, anthropometric trait and sex were included in each model (i.e., complete case analysis); therefore, sample sizes differ across ages. For interpretability, we present estimates on all measured units (e.g., kg/m^2^ and kg for BMI and weight, respectively); however, for the purposes of reference, we also present results for BMI on a standardized scale.

*MC4R* LoF produced by the mutations was analysed by comparing carriers of LoF mutations (i.e., all individuals with pLoF and cLoF mutations) to “non-LoF carriers” (i.e., all individuals with synonymous or common variants and no LoF mutation and those carrying WT-like or GoF mutations) as the reference group. Effect estimates therefore represent the mean difference in each anthropometric trait in carriers vs. non-carriers of *MC4R* LoF mutations. Associations were adjusted only for participant sex. We also assessed the effect of carriage of WT-like mutations on BMI, weight, and height compared to those with no detected LoF variant.

As *MC4R* mutation carriers have previously been reported to have lower BP than equivalently obese WT individuals, we additionally examined the association between *MC4R* LoF (defined in the same way as above) on clinically relevant obesity-related traits. Age-specific analyses were therefore conducted with measures of arterial and central BP and LVMI across the lifecourse, as proxies for cardiovascular health, both in a sex-adjusted model and a model additionally adjusted for BMI measured at the same occasion.

We also plotted the mean anthropometric trait at each time point, separating out carriers to the component mutational parts (i.e., comparing the reference “non-LoF carrier” group to pLoF and cLoF separately). Not all individuals with *MC4R* mutations had anthropometric/cardiovascular measurements available at all time points between birth and 24 years and, on some occasions, all LoF groups were not represented (e.g., no individuals carrying a cLoF mutation had anthropometric data at age 24 and many time points before the age of 5 had such data). In these instances, and for transparency, tables show results at all time points with all contributing individuals (and comment on the effect sizes and precision of these estimates) and figures only show results where all LoF mutational groups (pLoF and cLoF mutations) were represented by at least one individual (e.g., from 18 months to 18 years for BMI).

Sensitivity analyses were conducted to assess the effect of carrying vs. not carrying GoF mutations. Whilst there was an absence of a detectable effect, there was insufficient analytical power to assess these groups across a meaningful number of time points (results available on request). We also assessed the effect of excluding individuals carrying GoF mutations in main analyses, leaving only individuals with no LoF mutations and those carrying WT-like mutations in the “non-LoF carriers” reference group, but this made very little difference to findings (results available on request).

#### Longitudinal associations with anthropometric traits

Longitudinal analyses using linear-spline multi-level models were conducted to examine the association between the *MC4R* mutations and change in each anthropometric trait across the lifecourse. Given the limited number of WHR, fat mass and lean mass observations between birth and 24 years, longitudinal analyses focused on characterising the association between the *MC4R* mutations and BMI, weight, and height only. Additionally, given the lack of individuals carrying a cLoF mutation and anthropometric traits at age 24 years, longitudinal analyses were restricted to capture *MC4R*-driven anthropometric variation from the first instance that all LoF mutational groups were represented (i.e., 18 months for BMI and height, and birthweight) to 18 years of age. Multi-level models estimate the mean trajectories of each anthropometric trait, while accounting for non-independence of repeated measures within individuals, change in scale and variance of measures over time, and differences in the number and timing of measurements between individuals (using all available data from all eligible participants under a missing-at-random assumption). Linear splines allow knot points to be fitted at different ages to derive periods of change that are approximately linear. All participants with at least one measure of the anthropometric traits were included under a missing-at-random assumption to minimize selection bias in trajectories estimated using linear spline multi-level models (with two levels: measurement occasion and individual).

Knot points were placed as follows for each anthropometric trait based on the distribution and longitudinal pattern of measures between the earliest measure and 18 years: at ages 3.5, 5, 8 and 15 years for BMI; at ages 5 and 15 years for height; and at ages 1, 8 and 15 years for weight. Interaction terms between the variable indicating *MC4R* LoF (comprising the “non-LoF carrier” reference group (i.e., individuals with synonymous, common variations or no LoF mutation and individuals with GoF mutations) and carriers of LoF mutations) and each spline were included in the models to estimate the difference in the intercepts (earliest anthropometric trait measurement) and slopes (change in anthropometric trait from the earliest measure to 18 years across splines) between *MC4R* LoF. Additionally, interaction terms between sex and each spline were included to estimate the difference in intercepts and slopes between males and females; therefore, models were adjusted for sex.

### Sensitivity analyses

#### Comparison with β-arrestin-2 coupling

To understand the impact of β-arrestin-2-based *MC4R* functional classification on adiposity relative to those acting through cAMP signalling, we examined the age-specific associations between β-arrestin-2 LoF mutations with the same anthropometric traits as described above. Functional impairment of β-arrestin-2 coupling was coded in the same way as main analyses – comparing carriers (i.e., those carrying pLoF and cLoF mutations) to non-LoF carriers (i.e., individuals with synonymous, common variations or no LoF mutation and those with either WT-like or GoF mutations).

#### Comparison between rare and common variation

There has been growing interest in the use of the PRSs as predictors of phenotypes such as obesity and related metabolic fluctuations^28^. We assessed the comparative variation in anthropometric traits (namely, BMI) between carriage of *MC4R* LoF mutations with that of a genome-wide PRS comprising over 2 million common genetic variants weighted according to their effect sizes on BMI.

The weighted genome-wide PRS was generated in the same way as that used by Khera *et al*.^28^ and categorized as a binary variable reflecting individuals in the lower 90^th^ and upper 10^th^ percentiles of the PRS distribution. Age-specific analyses were conducted in the same way as above with all available measures of BMI and longitudinal models were performed on data capturing BMI from 18 months to 18 years of age, with groups of the PRS as the independent variable. For direct comparison with the effect sizes of the *MC4R* rare variation, these analyses were restricted to the individuals who were in the original sequenced set (n=5724).

To assess whether the genetic impact of the *MC4R* LoF mutations and the PRS were interconnected, we adjusted the main age-specific analyses of the association between *MC4R* LoF mutations and BMI for the genome-wide PRS at all available ages. We also calculated the variance in BMI explained by the PRS as compared to the *MC4R* LoF mutations using the measurement at age 18 years as an exemplar. We took the subset of participants who had both BMI measured at age 18 years and a derived PRS (N=3164, including 7 carriers) and ran a linear regression firstly with LoF mutation carrier status as the independent variable and secondly with the PRS as the independent variable (with BMI at age 18 years as the dependent variable in both cases). The R^2^ from these models was taken as an estimate of the variance explained in each case.

## Extended Data

## Supplementary Material

Supporting Information

## Figures and Tables

**Table 1:** Sanger sequencing validated non-synonymous *MC4R* mutations identified in those sequenced in ALSPAC

*MC4R* variant	Genomic position (bp)	Codon change	Database accession(s)	Domain	No of carriers^1^	ALSPAC MAF
p.T5N^2^	Chr18:60372336	aCc/aAc	rs752432398	N-term	1	0.0087%
p.T11A	Chr18:60372319	Act/Gct	rs372794914	N-term	2	0.0175%
p.S30F	Chr18:60372261	tCc/tTc	rs13447323	N-term	3	0.0262%
p.S36T	Chr18:60372244	Tct/Act	rs954123325	N-term	1	0.0087%
p.T53I	Chr18:60372192	aCt/aTt	rs141148170	TM1	3	0.0262%
p.Y80C	Chr18:60372111	tAc/tGc	rs1368643838	TM2	1	0.0087%
p.S85I^2^	Chr18:60372096	aGc/aTc	rs1420993856	TM2	1	0.0087%
p.V95I	Chr18:60372067	Gtt/Att	rs13447328	TM2	1	0.0087%
p.V103I^3^	Chr18:60372043	Gtc/Atc	rs2229616	TM2	-	-
p.T112M	Chr18:60372015	aCg/aTg	rs13447329	ECL1	4	0.0349%
p.N123S^2^	Chr18:60371982	aAt/aGt	rs761982475	TM3	1	0.0087%
p.S127L	Chr18:60371970	tCg/tTg	rs13447331	TM3	1	0.0087%
p.I137T	Chr18:60371940	aTt/aCt	rs151102515	TM3	2	0.0173%
p.H158R	Chr18:60371877	cAt/cGt	rs202081467	ICL2	2	0.0173%
p.S180P	Chr18:60371812	Tca/Cca	rs193922685	TM4	1	0.0087%
p.F184L^2^	Chr18:60371800	Ttc/Ctc	-	TM4	1	0.0087%
p.F202L	Chr18:60371744	ttC/ttA	rs138281308	TM5	2	0.0175%
p.M215I	Chr18:60371705	atG/atA	rs768687497	TM5	1	0.0087%
p.A227T^2^	Chr18:60371671	Gct/Act	rs201736647	ICL3	1	0.0087%
p.R236C	Chr18:60371644	Cgc/Tgc	rs758426526	ICL3	1	0.0087%
p.G238V*fs*X4^2^	Chr18:60371636-60371637	gGt/gt	-	TM6	1	0.0087%
p.N240S	Chr18:60371631	aAt/aGt	rs202228712	TM6	2	0.0175%
p.I251W*fs*X34	Chr18:60371598-60371600	ctGAtt/cttt	rs13447339	TM6	1	0.0087%
p.I251L^3^	Chr18:60371599	Att/Ctt	rs52820871	TM6	-	-
p.G252S	Chr18:60371596	Ggc/Agc	rs13447336	TM6	1	0.0087%
p.V253I	Chr18:60371593	Gtc/Atc	rs187152753	TM6	2	0.0175%
p.C271Y	Chr18:60371538	tGt/tAt	rs121913562	ECL3	1	0.0087%
p.S295P	Chr18:60371467	Tca/Cca	rs368264587	TM7	1	0.0087%
p.G323V^2^	Chr18:60371382	gGa/gTa	rs926626133	C-term	1	0.0087%

*ALSPAC = Avon Longitudinal Study of Parents and Children; BP = base-pair position; C-term = C-terminus; ECL = extracellular loop; MAF = minor allele frequency; N-term = N-terminus; TM = transmembrane*.

1 Some mutations listed were carried by multiple individuals.

2 Previously uncharacterised functionally.

3 The common variants, p.V103I and p.I251L, were excluded from the main analyses.

**Table 2:** Prevalence estimates of *MC4R* LoF of cAMP accumulation identified in the ALSPAC sample used for analyses

Mutational characterisation	Frequency	Prevalence estimate (% of total)
Synonymous, common variations or no LoF mutation	5684	99.30
GoF	2	0.03
WT-like	21	0.37
pLoF	13	0.23
cLoF	4	0.07
**Total**	**5724**	**100**

*ALSPAC = Avon Longitudinal Study of Parents and Children; cLoF = complete loss of function; GoF = gain of function; LoF = loss of function; pLoF = partial loss of function; SD = standard deviation; WT = wild-type*.

## Data Availability

Full details of the cohort and study design have been described previously and are available at http://www.alspac.bris.ac.uk. Please note that the study website contains details of all the data that is available through a fully searchable data dictionary and variable search tool (http://www.bristol.ac.uk/alspac/researchers/our-data/). ALSPAC data are available through a system of managed open access. Data for this project was accessed under the project number B2891. The application steps for ALSPAC data access are: Please read the ALSPAC access policy which describes the process of accessing the data in detail, and outlines the costs associated with doing so. You may also find it useful to browse the fully searchable research proposals database, which lists all research projects that have been approved since April 2011. Please submit your research proposal for consideration by the ALSPAC Executive Committee. You will receive a response within 10 working days to advise you whether your proposal has been approved. If you have any questions about accessing data, please email alspac-data@bristol.ac.uk.

## Code Availability

Code for data analyses is freely available on request
