# Supplementary material for: Loss of function mutations in the melanocortin 4 receptor in a UK birth cohort
Source: Nat Med. Author manuscript; Available in PMC 2021 Oct 14. (PMC7611835; doi:10.1038/s41591-021-01349-y)
Supplement: Supporting Information [file EMS136341-supplement-Supporting_Information.pdf]

Supplementary Table 1. Differences in characteristics in those sequenced for *MC4R* LoF mutations and those not sequenced from the rest of ALSPAC.

Trait	Included		Excluded		Difference	
	N	Mean (SD) or %	N	Mean (SD) or %	Mean (SD) or %	P-value[1]
Parental characteristics						
Maternal age at birth of first child	5317	25.49 (4.84)	7835	23.55 (4.95)	1.94 (0.09)	1.42×10^{-109}
Maternal pre-pregnancy BMI	4893	22.91 (3.74)	6621	22.95 (3.93)	-0.03 (0.07)	0.63
Maternal weight gain in pregnancy	4919	12.63 (4.55)	7282	12.51 (4.69)	0.12 (0.09)	0.16
Parity	5236	0.80 (0.92)	7695	0.87 (1.06)	-0.07 (0.02)	3.62×10^{-05}
Highest household social class	5,137		7,124			2.08×10^{-58}
Professional (I)	582	11.33	514	7.22	4.11	
Managerial and technical (II)	2,161	42.07	2,398	33.66	8.41	
Skilled, non-manual (III)	1,506	29.32	2,269	31.85	-2.53	
Skilled, manual (III)	556	10.82	1,119	15.71	-4.89	
Partly skilled (IV)	280	5.45	647	9.08	-3.63	
Unskilled (V)	52	1.01	177	2.48	-1.47	
Family income (£/wk)	4353		4365			1.58×10^{-35}
<100	249	5.72	516	11.82	-6.10	
100-199	657	15.09	882	20.21	-5.12	
200-299	1,256	28.85	1,217	27.88	0.97	
300-399	1,023	23.50	827	18.95	4.55	
>400	1,168	26.83	923	21.15	5.68	
Maternal education at 33 months	5,041		6,505			2.31×10^{-79}
CSE	493	9.78	1,231	18.92	-9.14	
Vocational	426	8.45	787	12.10	-3.65	
O-level	1,836	36.42	2,427	37.31	-0.89	
A-level	1,401	27.79	1,359	20.89	6.90	
Degree	885	17.56	701	10.78	6.78	
Paternal education at 33 months	4,781		5,838			9.22×10^{-27}
CSE	688	14.39	1,192	20.42	-6.03	
Vocational	407	8.51	594	10.17	-1.66	
O-level	1,123	23.49	1,390	23.81	-0.32	
A-level	1,426	29.83	1,651	28.28	1.55	
Degree	1,137	23.78	1,011	17.32	6.46	
Participant characteristics						
Continued education at 18 years (% yes)	2902	88.15	1147	87.36	0.79	0.49
Sex (% female)	5717	48.52	13543	48.38	0.14	0.86
Ethnicity (% white)	5150	96.08	6820	94.11	1.97	1.04×10^{-06}

ALSPAC = Avon Longitudinal Study of Parents of Children; BMI = body mass index; CSE = Certificate of Secondary Education; SD = standard deviation.

[1]For continuous variables, P-values were derived from a t-test of the mean difference between those included vs. excluded from the group of sequenced individuals. For binary and ordered categorical variables, P-values were derived from a t-test of the mean difference in trend across all categories. All p-values were uncorrected for multiple comparisons.

Genomic location (GRCh38)	Reference allele	Consequence	CDS position	Codon substitution	Protein position	Amino acid substitution	RSID	MAF (1000 Genomes)	MAF (gnomAD)*	EC50 (WT-fold)**	logEC50 Diff p-value (N) ***	EC50 classification	Emax (% WT)**	Emax Diff p-value (N) ***	Emax classification	Overall LoF Class	Reference
18:60372336-60372336	T	missense_variant	14	aCc/aAc	5	T/N	rs752432398	0	0.000008209	1.30	0.62 (5)	WT-Like	96.0	0.73 (5)	WT-Like	WT-like	
18:60372319-60372319	C	missense_variant	31	Act/Gct	11	T/A	rs372794914	0	0.00005721	0.48, 1,53		WT-Like	109.0		WT-Like	WT-like	1,2
18:60372261-60372261	A	missense_variant	89	tCc/tTc	30	S/F	rs13447323	0	0.00008533	2.3, *0.45 , 1.25 , 1.98*		WT-Like	*157.1 , 72.9 , 80*		WT-Like	WT-like	2, 3, 4,5
18:60372244-60372244	T	missense_variant	106	Tct/Act	36	S/T	rs954123325	0	0.000004063	1.28		WT-Like	88.0		WT-Like	WT-like	6
18:60372192-60372192	A	missense_variant	158	aCt/aTt	53	T/I	rs141148170	0	0.000008126	*0.68*		WT-Like	*77.2*		WT-Like	WT-like	7
18:60372111-60372111	C	missense_variant	239	tAc/tGc	80	Y/C	rs1368643838	0	0	*7.34*		pLoF	*39.8*		pLoF	pLoF	7
18:60372096-60372096	A	missense_variant	254	aGc/aTc	85	S/I	rs1420993856	0	0	-	Not determined	-	-0.6	1.60e-5 (5)	cLoF	cLoF	
18:60372067-60372067	T	missense_variant	283	Gtt/Att	95	V/I	rs13447328	0.0002	0.00003655	*1.76, 3.01*		WT-Like	*17.3, 94, 0, 47.6*		pLoF	pLoF	3, 8, 2, 7
18:60372043-60372043	T	missense_variant	307	Gtc/Atc	103	V/I	rs2229616	0.0162	0.01614	1.00, 0.71, 2.03,1.11		WT-Like	90.0, 105.2, 165		WT-Like/GoF	WT-Like/GoF	9, 10, 2, 11
18:60372015-60372015	A	missense_variant	335	aCg/aTg	112	T/M	rs13447329	0	0.000918	1.00, 0.72, 0.37		WT-Like	90, 120		WT-Like	WT-like	9, 1, 2
18:60371982-60371982	C	missense_variant	368	aAt/aGt	123	N/S	rs761982475	0	0.00002843	2.60	0.25 (5)	WT-Like	79.0	0.28 (5)	WT-Like	WT-like	
18:60371970-60371970	A	missense_variant	380	tCg/tTg	127	S/L	rs13447331	0	0.0001706	0.93, 1,46		WT-Like	57.0		pLoF	pLoF	2, 12
18:60371940-60371940	G	missense_variant	410	aTt/aCt	137	I/T	rs151102515	0.0002	0.00002031	5.01, 4.0		WT-Like	65.0		pLoF	pLoF	9, 2
18:60371877-60371877	C	missense_variant	473	cAt/cGt	158	H/R	rs202081467	0	0.000004063	0.37		WT-Like	135.0		GoF	GoF	13
18:60371812-60371812	G	missense_variant	538	Tca/Cca	180	S/P	rs193922685	0	0.000004063	*7.84*		pLoF	*79.3*		WT-Like	pLoF	7
18:60371800-60371800	G	missense_variant	550	Ttc/Ctc	184	F/L		0	0	8.60	0.0003 (5)	pLoF	71.0	0.07 (5)	WT-Like	pLoF	
18:60371744-60371744	T	missense_variant	606	ttC/ttA	202	F/L	rs138281308	0.0028	0.0006502	1.08, 0.83, 1.57, 1.34		WT-Like	89.0, 83.0, 126.0		WT-Like	WT-like	1, 6, 14, 15
18:60371705-60371705	T	missense_variant	645	atG/atA	215	M/I	rs768687497	0	0.000008129	-		-	*31.0*		pLoF	pLoF	7
18:60371671-60371671	T	missense_variant	679	Gct/Act	227	A/T	rs201736647	0	0.000004065	2.20	0.27 (5)	WT-Like	90.0	0.46 (5)	WT-Like	WT-like	
18:60371644-60371644	A	missense_variant	706	Cgc/Tgc	236	R/C	rs758426526	0	0.00006908	*0.21*		WT-Like	*90.2*		WT-Like	WT-like	4
18:60371637-60371637	-C	frameshift_variant	713	gGt/gt	238	G/X		0	0	-	Not determined	-	0.0	7.59e-6 (3)	cLoF	cLoF	
18:60371631-60371631	C	missense_variant	719	aAt/aGt	240	N/S	rs202228712	0.0008	0.0001666	0.48, 0.52		WT-Like	72.0		pLoF	pLoF	1, 14
18:60371599-60371600	-TC	frameshift_variant	750-751	ctGAtt/cttt	250-251	LI/LX	rs13447339	0	0.0000358	-		-	*0.0*		cLoF	cLoF	14
18:60371599-60371599	G	missense_variant	751	Att/Ctt	251	I/L	rs52820871	0.0026	0.006867	0.83, *1.52, 3.17, 1.13, 0.71, 0.60*		WT-Like	*120.0, 100.3, 110.3, 100.0*		WT-Like	WT-Like	2, 16, 17, 18, 19
18:60371596-60371596	T	missense_variant	754	Ggc/Agc	252	G/S	rs13447336	0	0.00003657	2.7, *3.17, 0.73, 6.21, 9.40, 7.00, 6.38*		pLoF	*80.0, 99.0, 107.0, 73.7, 57.2*		WT-Like	pLoF	2, 5, 3, 8, 20, 21
18:60371593-60371593	T	missense_variant	757	Gtc/Atc	253	V/I	rs187152753	0.0006	0.00006501	0.87, *3.51, 1.00, 0.08, 1.06, 0.92, 2.61*		WT-Like	*90.0, 60.0, 55.0, 92.0, 59.8*		pLoF	pLoF	2, 5, 22, 23, 24,7
18:60371538-60371538	T	missense_variant	812	tGt/tAt	271	C/Y	rs121913562	0	0	58.00		cLoF	20.0		cLoF	cLoF	2, 25
18:60371467-60371467	G	missense_variant	883	Tca/Cca	295	S/P	rs368264587	0	0.00001219	1.48, 2.91		WT-Like	112.0		WT-Like	WT-like	1, 13
18:60371382-60371382	A	missense_variant	968	gGa/gTa	323	G/V	rs926626133	0	0.00001219	0.80	0.78 (5)	WT-Like	84.0	0.10 (5)	WT-Like	WT-like	

missense_variant common variants excluded from main analysis
missense_variant previously characterised for cAMP activity
missense_variant new, uncharactersied variant

Functional Classification	cLoF	pLoF	WT-Like	GoF
**EC50 (WT-fold)**	>25X	>5X and ≤25X	≥0.2X and ≤	<0.2X
**Emax (%WT)**	<25%	≥25% and <75%	≥75% and ≥1	>125%

*gnomAD MAF estimations are for all populations combined.
**NDP-alphaMSH EC50 and EMax are adopted from Collet et al 2017 and Lotta et al 2019, alphaMSH values used when NDP-alphaMSH data is not complete (*italicised*). Values from multiple references are quoted in the table.
*** P-values and N are only available for variants functionally characterised in this study. Difference for each variant was compared to MC4R Wild-type, N=6. Bonferroni cutoff for logEC50 = 0.01 and Emax = 0.007
CDS = coding sequence; MAF = minor allele frequency

References

1 Tao, Y.-X. & Segaloff, D. L. *Endocrinology* **144**, 4544–4551 (2003).
2 Xiang, Z. *et al.* *Biochemistry* **45**, 7277–7288 (2006).
3 Hinney, A. *et al.* *J. Clin. Endocrinol. Metab.* **88**, 4258–4267 (2003).
4 Calton, M. A. *et al. Hum. Mol. Genet.* **18**, 1140–1147 (2009).
5 Lubrano-Berthelier, C. *et al* . *Hum. Mol. Genet.* **12**, 145–153 (2003).
6 Hughes, D. A. *et al.* *Hum. Genet.* **124**, 633–647 (2009).
7 Collet, T.-H. *et al.* *Mol. Metab.* **6**, 1321–1329 (2017).
8 Lubrano-Berthelier, C. *et al. J. Clin. Endocrinol. Metab.* **91**, 1811–1818 (2006).
9 Gu, W. *et al.* *Diabetes* **48**, 635–639 (1999).
10 Ho, G. & MacKenzie, R. G. *J. Biol. Chem.* **274**, 35816–35822 (1999).
11 Lotta, L. A. *et al.* *Cell* **177**, 597-607.e9 (2019).
12 Fan, Z.-C. & Tao, Y.-X. *J. Cell. Mol. Med.* **13**, 3268–3282 (2009).
13 Hinney, A. *et al.* *J. Clin. Endocrinol. Metab.* **91**, 1761–1769 (2006).
14 Xiang, Z., Proneth, B., Dirain, M. L., Litherland, S. A. & Haskell-Luevano, C. *Biochemistry* **49**, 4583–4600 (2010).
15 Hohenadel, M. G. *et al.* *Int. J. Obes. (Lond).* **38**, 1068–1074 (2014).
16 Vaisse, C. *et al.* *J. Clin. Invest.* **106**, 253–262 (2000).
17 Thearle, M. S. *et al.* *Diabetes* **61**, 250–257 (2012).
18 Melchior, C. *et al.* *Horm. Res. Paediatr.* **78**, 237–246 (2012).
19 Rovite, V. *et al.* *Mol. Biol. Rep.* **41**, 1491–1500 (2014).
20 Roubert, P. *et al.* *J. Endocrinol.* **207**, 177–183 (2010).
21 Hatoum, I. J. *et al.* *J. Clin. Endocrinol. Metab.* **97**, E1023-31 (2012).
22 Yeo, G. S. H. *et al.* *Hum. Mol. Genet.* **12**, 561–574 (2003).
23 Farooqi, I. S. *et al.* *N. Engl. J. Med.* **348**, 1085–1095 (2003).
24 Nijenhuis, W. A. J., Garner, K. M., van Rozen, R. J. & Adan, R. A. H. *J. Biol. Chem.* **278**, 22939–22945 (2003).
25 Tao, Y.-X. & Segaloff, D. L. *J. Clin. Endocrinol. Metab.* **90**, 5632–5638 (2005).

Supplementary Table 3. List of all *MC4R* mutations grouped by their cAMP LoF classification and their corresponding β-arrestin-2 classification.

**Mutation**	**cAMP classification**	**β-arrestin-2 classification**
**p.S85I**	cLoF	cLoF
**p.G238V*fs*X4**	cLoF	cLoF
**p.I251W*fs*X40**	cLoF	cLoF
**p.C271Y**	cLoF	cLoF
**p.Y80C**	pLoF	cLoF
**p.V95I**	pLoF	pLoF
**p.S127L**	pLoF	pLoF
**p.I137T**	pLoF	pLoF
**p.S180P**	pLoF	pLoF
**p.V253I**	pLoF	pLoF
**p.F184L**	pLoF	WT-Like
**p.M215I**	pLoF	WT-Like
**p.N240S**	pLoF	GoF
**p.G252S**	pLoF	WT-Like
p.T5N	WT-like	WT-Like
p.S30F	WT-like	WT-Like
p.S36T	WT-like	WT-Like
p.T112M	WT-like	WT-Like
p.N123S	WT-like	WT-Like
p.F202L	WT-like	WT-Like
p.A227T	WT-like	WT-Like
p.R236C	WT-like	WT-Like
p.S295P	WT-like	pLoF
p.G323V	WT-like	WT-Like
p.T11A	WT-like	GoF
p.T53I	WT-like	GoF
p.H158R	GoF	GoF

cAMP = cyclic adenosine monophosphate; cLoF = complete loss of function; GoF = gain of function; LoF = loss of function; pLoF = partial loss of function; WT-like = wild-type like. Individuals with any of the 14 LoF mutations marked in bold were those that comprised the "carrier" group (i.e., combining both cLoF and pLoF mutations) in main analyses of the association between MC4R LoF and anthropometric traits. These were compared to the reference group (i.e., non-LoF carriers - comprising individuals with synonymous, common variations or no LoF mutation and individuals with any of the listed (non-bold) GoF or WT-like mutations).

Supplementary Table 4. Age-specific associations between *MC4R* LoF of cAMP accumulation and BMI, weight and height.

Age	BMI (kg/m^2)			Weight (kg)			Height (cm)		
	**N (ref, pLoF, cLoF)[1]**	**Effect estimate (95% CI)[2]**	**P**	**N (ref, pLoF, cLoF)[1]**	**Effect estimate (95% CI)[2]**	**P**	**N (ref, pLoF, cLoF)[1]**	**Effect estimate (95% CI)[2]**	**P**
Birth				5354 (5338; 12; 4)	0.22 (-0.04, 0.48)	0.10			
4 months	967 (966; 1; 0)	-0.15 (-3.09, 2.80)	0.92	1034 (1031; 3; 0)	1.08 (0.21, 1.95)	0.01	970 (969; 1; 0)	0.97 (-3.80, 5.74)	0.69
8 months	3307 (3300; 7; 0)	0.19 (-0.96, 1.33)	0.75	3690 (3682; 8; 0)	0.34 (-0.35, 1.02)	0.34	3375 (3368; 7; 0)	0.16 (-1.80, 2.11)	0.87
12 months	1370 (1368; 2; 0)	1.79 (-0.26, 3.84)	0.09	1613 (1609; 3; 1)	1.70 (0.59, 2.82)	0.003	1386 (1384; 2; 0)	-0.72 (-4.56, 3.13)	0.71
18 months	2719 (2714; 3; 2)	1.20 (-0.14, 2.53)	0.08	2905 (2900; 3; 2)	1.45 (0.35, 2.55)	0.01	2797 (2792; 3; 2)	1.84 (-0.86, 4.55)	0.18
2 years	891 (890; 1; 0)	-0.17 (-2.98, 2.63)	0.90	925 (924; 1; 0)	0.90 (-1.92, 3.71)	0.53	913 (912; 1; 0)	3.49 (-2.64, 9.63)	0.26
2.5 years	673 (672; 1; 0)	-0.45 (-3.16, 2.26)	0.74	698 (697; 1; 0)	0.99 (-2.22, 4.20)	0.55	681 (680; 1; 0)	4.71 (-1.67, 11.10)	0.15
3 years	1052 (1051; 1; 0)	-0.38 (-3.22, 2.47)	0.80	1081 (1080; 1; 0)	0.96 (-2.53, 4.45)	0.59	1097 (1096; 1; 0)	4.32 (-2.79, 11.42)	0.23
3.5 years	2443 (2436; 5; 2)	0.32 (-0.69, 1.33)	0.54	2522 (2515; 5; 2)	0.99 (-0.40, 2.39)	0.16	2527 (2518; 6; 3)	3.25 (0.79, 5.71)	0.01
4 years	1111 (1109; 2; 0)	-0.43 (-2.46, 1.60)	0.67	1131 (1129; 2; 0)	0.47 (-2.54, 3.48)	0.76	1154 (1152; 2; 0)	2.89 (-2.74, 8.52)	0.31
5 years	1284 (1281; 2; 1)	1.55 (-0.45, 3.54)	0.13	1321 (1318; 2; 1)	3.92 (0.70, 7.14)	0.02	1383 (1380; 2; 1)	4.66 (-1.34, 10.66)	0.13
8 years	5243 (5228; 11; 4)	2.92 (1.90, 3.94)	1.99x10^{-08}	5243 (5228; 11; 4)	6.55 (4.21, 8.90)	4.54x10^{-08}	5248 (5233; 11; 4)	3.67 (0.79, 6.56)	0.01
9 years	4452 (4439; 9; 4)	3.83 (2.56, 5.10)	3.59x10^{-09}	4560 (4547; 9; 4)	9.65 (6.54, 12.75)	1.25x10^{-09}	4715 (4701; 10; 4)	4.51 (1.46, 7.56)	0.004
10 years	5378 (5363; 11; 4)	4.08 (2.66, 5.51)	1.99x10^{-08}	5412 (5397; 11; 4)	11.54 (7.83, 15.24)	1.10x10^{-09}	5381 (5366; 11; 4)	5.34 (2.13, 8.54)	0.001
11 years	5050 (5034; 13; 3)	3.32 (1.84, 4.81)	1.18x10^{-05}	5084 (5068; 13; 3)	9.76 (5.72, 13.81)	2.27x10^{-06}	5059 (5043; 13; 3)	4.46 (1.20, 7.72)	0.01
12 years	4929 (4914; 12; 3)	3.93 (2.25, 5.61)	4.67x10^{-06}	4934 (4919; 12; 3)	13.48 (8.52, 18.43)	1.01x10^{-07}	4931 (4916; 12; 3)	6.53 (2.88, 10.17)	4.50x10^{-04}
13 years	4650 (4636; 11; 3)	4.64 (2.85, 6.43)	4.00x10^{-07}	4650 (4636; 12; 3)	15.59 (10.01, 21.18)	4.70x10^{-08}	4695 (4681; 11; 3)	5.93 (1.91, 9.95)	0.004
14 years	4352 (4340; 9; 3)	3.68 (1.74, 5.62)	2.04x10^{-04}	4352 (4340; 9; 3)	14.05 (7.70, 20.41)	1.50x10^{-05}	4357 (4345; 9; 5)	5.55 (1.21, 9.90)	0.01
15 years	3866 (3853; 10; 3)	4.61 (2.75, 6.46)	1.23x10^{-06}	3866 (3853; 10; 3)	17.32 (11.15, 23.5)	3.99x10^{-08}	3872 (3859; 10; 3)	4.50 (0.76, 8.24)	0.02
18 years	3499 (3490; 7; 2)	4.84 (2.19, 7.49)	3.42x10^{-04}	3501 (3492; 7; 2)	17.76 (9.41, 26.10)	3.11x10^{-05}	3502 (3493; 7; 2)	3.17 (-1.08, 7.42)	0.14
24 years	2695 (2692; 3; 0)	1.71 (-3.79, 7.20)	0.54	2697 (2694; 3; 0)	10.93 (-6.20, 28.06)	0.21	2697 (2694; 3; 0)	5.77 (-1.43, 12.96)	0.12

BMI = body mass index; CI = confidence interval; cLoF = complete loss of function; GoF = gain of function; LoF = loss of function; pLoF = partial loss of function; SD = standard deviation; WT = wild-type. [1]N represents the total sample size in each analysis with numbers in brackets representing the number of individuals in the reference group (i.e., individuals with synonymous, common variations or no LoF mutation, and individuals with GoF or WT-like mutations – the "non-LoF carriers"), and individuals with pLoF or cLoF mutations, respectively. [2]Estimates represent the change in each anthropometric trait (units in table) in carriers vs. non-carriers of MC4R LoF mutations. Analyses were linear regression models adjusted for sex, with p-values uncorrected for multiple comparisons.

Supplementary Table 5. Age-specific associations between *MC4R* LoF of cAMP accumulation and standardized BMI.

Age	BMI (SD)		
	**N (ref, pLoF, cLoF)**[1]	**Effect estimate (95% CI)**[2]	**P**
4 months	967 (966; 1; 0)	-0.09 (-2.01, 1.83)	0.93
8 months	3307 (3300; 7; 0)	0.12 (-0.62, 0.85)	0.75
12 months	1370 (1368; 2; 0)	1.18 (-0.15, 2.52)	0.08
18 months	2719 (2714; 3; 2)	0.80 (-0.06, 1.66)	0.07
2 years	891 (890; 1; 0)	-0.12 (-2.09, 1.84)	0.90
2.5 years	673 (672; 1; 0)	-0.34 (-2.32, 1.64)	0.74
3 years	1052 (1051; 1; 0)	-0.26 (-2.15, 1.62)	0.78
3.5 years	2443 (2436; 5; 2)	0.26 (-0.47, 0.98)	0.49
4 years	1111 (1109; 2; 0)	-0.32 (-1.72, 1.98)	0.66
5 years	1284 (1281; 2; 1)	0.90 (-0.22, 2.02)	0.11
8 years	5243 (5228; 11; 4)	1.45 (0.97, 1.93)	3.64×10^{-09}
9 years	4452 (4439; 9; 4)	1.64 (1.12, 2.16)	8.25×10^{-10}
10 years	5378 (5363; 11; 4)	1.46 (0.97, 1.95)	6.25×10^{-09}
11 years	5050 (5034; 13; 3)	1.08 (0.61, 1.55)	7.12×10^{-06}
12 years	4929 (4914; 12; 3)	1.17 (0.68, 1.65)	3.01×10^{-06}
13 years	4650 (4636; 11; 3)	1.33 (0.82, 1.84)	2.90×10^{-07}
14 years	4352 (4340; 9; 3)	1.07 (0.52, 1.63)	1.57×10^{-04}
15 years	3866 (3853; 10; 3)	1.31 (0.79, 1.83)	9.11×10^{-07}
18 years	3499 (3490; 7; 2)	1.18 (0.55, 1.81)	2.24×10^{-04}
24 years	2695 (2692; 3; 0)	0.38 (-0.70, 1.47)	0.49

BMI = body mass index; CI = confidence interval; cLoF = complete loss of function; GoF = gain of function; LoF = loss of function; pLoF = partial loss of function; SD = standard deviation; WT = wild-type. [1]N represents the total sample size in each analysis with numbers in brackets representing the number of individuals in the reference group (i.e., individuals with synonymous, common variations or no LoF mutation, and individuals with GoF or WT-like mutations – the "non-LoF carriers"), and individuals with pLoF or cLoF mutations, respectively. [2]Estimates represent the SD change in BMI in carriers vs. non-carriers of MC4R LoF mutations. Analyses were linear regression models adjusted for sex, with p-values uncorrected for multiple comparisons.

Supplementary Table 6. Age-specific associations between *MC4R* LoF of cAMP accumulation and WHR, fat mass and lean mass.

Age	Fat mass (kg)			Lean mass(kg)			WHR		
	N (ref, pLoF, cLoF)[1]	Effect estimate (95% CI)[2]	P	N (ref, pLoF, cLoF)[1]	Effect estimate (95% CI)[2]	P	N (ref, pLoF, cLoF)[1]	Effect estimate (95% CI)[2]	P
8 years							5078 (5063; 11; 4)	0.01 (-0.01, 0.03)	0.48
10 years	5109 (5095; 10; 4)	8.16 (5.59, 10.72)	4.71×10^{-10}	5109 (5095; 10; 4)	3.24 (1.63, 4.85)	8.06×10^{-05}	5339 (5324; 11; 4)	0.04 (0.02, 0.06)	0.001
12 years	4874 (4859; 12; 3)	8.64 (5.33, 11.96)	3.39×10^{-07}	4874 (4859; 12; 3)	4.28 (2.08, 6.48)	1.38×10^{-04}	4926 (4911; 12; 3)	0.04 (0.01, 0.06)	0.01
14 years	4295 (4283; 9; 3)	9.76 (5.49, 14.04)	7.86×10^{-06}	4295 (4283; 9; 3)	3.81 (0.49, 7.14)	0.02			
15 years	3750 (3737; 10; 3)	12.34 (7.91, 16.78)	5.27×10^{-08}	3750 (3737; 10; 3)	4.04 (1.04, 7.04)	0.01			
18 years	3408 (3399; 7; 2)	14.78 (8.56, 20.99)	3.27×10^{-06}	3408 (3399; 7; 2)	2.00 (-1.48, 5.47)	0.26			
24 years	2631 (2628; 3; 0)	9.08 (-2.67, 20.84)	0.13	2631 (2628; 3; 0)	1.58 (-5.58, 8.74)	0.67	2692 (2689; 3; 0)	0.04 (-0.03, 0.10)	0.29

CI = confidence interval; cLoF = complete loss of function; GoF = gain of function; LoF = loss of function; pLoF = partial loss of function; SD = standard deviation; WHR = waist-hip ratio WT = wild-type.

[1]N represents the total sample size in each analysis with numbers in brackets representing the number of individuals in the reference group (i.e., individuals with synonymous, common variations or no LoF mutation and individuals with GoF or WT-like mutations – the "non-LoF carriers"), and individuals with pLoF or cLoF mutations, respectively. [2]Estimates represent the change in each anthropometric trait (units in table) in carriers vs. non-carriers of MC4R LoF mutations. Analyses were linear regression models adjusted for sex, with p-values uncorrected for multiple comparisons.

Supplementary Table 7. Age-specific associations showing mean differences in BMI, weight and height comparing individuals with WT-like mutations for cAMP accumulation and non-LoF carriers not carrying WT-like mutations.

Age	BMI (kg/m²)			Weight (kg)			Height (cm)		
	N (ref, WT-like)[1]	Effect estimate (95% CI)[2]	P	N (ref, WT-like)[1]	Effect estimate (95% CI)[2]	P	N (ref, WT-like)[1]	Effect estimate (95% CI)[2]	P
Birth				5336 (5315; 21)	-0.09 (-0.32, 0.14)	0.45			
4 months	966 (962; 4)	0.62 (-0.86, 2.09)	0.41	1031 (1027; 4)	-0.02 (-0.78, 0.73)	0.95	969 (965; 4)	-1.12 (-3.51, 1.27)	0.36
8 months	3297 (3281; 16)	-0.08 (-0.84, 0.68)	0.84	3682 (3665; 17)	-0.20 (-0.67, 0.28)	0.42	3365 (3348; 17)	-0.44 (-1.70, 0.81)	0.49
12 months	1366 (1359; 7)	-0.05 (-1.15, 1.05)	0.93	1608 (1600; 8)	0.17 (-0.62, 0.95)	0.68	1382 (1375; 7)	0.77 (-1.27, 2.82)	0.46
18 months	2713 (2698; 15)	0.43 (-0.34, 1.20)	0.28	2899 (2883; 16)	0.001 (-0.61, 0.62)	0.996	2791 (2776; 15)	-0.60 (-2.16, 0.97)	0.45
2 years	890 (886; 4)	1.14 (-0.26, 2.55)	0.11	924 (920; 4)	0.75 (-0.66, 2.16)	0.30	912 (908; 4)	-0.40 (-3.47, 2.67)	0.80
2.5 years	672 (669; 3)	0.34 (-1.23, 1.91)	0.67	697 (694; 3)	0.37 (-1.49, 2.22)	0.70	680 (677; 3)	0.40 (-3.30, 4.09)	0.83
3 years	1051 (1048; 3)	0.35 (-1.30, 2.00)	0.68	1080 (1076; 4)	0.09 (-1.66, 1.84)	0.92	1096 (1093; 3)	-0.52 (-4.62, 3.58)	0.80
3.5 years	2434 (2423; 11)	-0.40 (-1.21, 0.40)	0.33	2513 (2502; 11)	-0.33 (-1.44, 0.78)	0.56	2517 (2506; 11)	0.16 (-2.05, 2.38)	0.89
4 years	1109 (1105; 4)	0.19 (-1.26, 1.64)	0.80	1129 (1125; 4)	0.06 (-2.07, 2.20)	0.95	1152 (1148; 4)	-0.53 (-4.51, 3.45)	0.79
5 years	1281 (1276; 5)	-0.23 (-1.78, 1.32)	0.77	1318 (1312; 6)	-0.75 (-3.03, 1.52)	0.52	1380 (1375; 5)	-1.89 (-6.54, 2.76)	0.43
8 years	5226 (5208; 18)	-0.64 (-1.57, 0.29)	0.17	5226 (5208; 18)	-1.98 (-4.11, 0.16)	0.07	5231 (5213; 18)	-2.36 (-4.99, 0.28)	0.08
9 years	4437 (4419; 18)	-0.49 (-1.57, 0.59)	0.37	4545 (4527; 18)	-1.55 (-4.19, 1.08)	0.25	4699 (4681; 18)	-1.68 (-4.37, 1.01)	0.22
10 years	5360 (5339; 21)	-0.79 (-1.99, 0.41)	0.20	5395 (5374; 21)	-2.16 (-5.29, 0.96)	0.18	5363 (5342; 21)	-1.13 (-3.84, 1.57)	0.41
11 years	5032 (5014; 18)	-0.42 (-1.82, 0.98)	0.56	5066 (5048; 18)	-1.94 (-5.74, 1.87)	0.32	5041 (5023; 18)	-1.83 (-4.90, 1.24)	0.24
12 years	4913 (4895; 18)	-0.17 (-1.70, 1.37)	0.83	4918 (4900; 18)	-1.23 (-5.75, 3.29)	0.59	4915 (4897; 18)	-1.07 (-4.39, 2.26)	0.53
13 years	4635 (4618; 17)	-0.09 (-1.72, 1.53)	0.91	4635 (4618; 17)	-0.98 (-6.04, 4.09)	0.71	4680 (4663; 17)	-0.84 (-4.48, 2.81)	0.65
14 years	4339 (4321; 18)	0.03 (-1.55, 1.61)	0.97	4339 (4321; 18)	0.20 (-4.99, 5.39)	0.94	4344 (4326; 18)	0.47 (-3.07, 4.02)	0.79
15 years	3852 (3837; 15)	-0.37 (-2.10, 1.36)	0.68	3852 (3837; 15)	-0.89 (-6.63, 4.85)	0.76	3858 (3843; 15)	0.01 (-3.46, 3.49)	0.99
18 years	3489 (3478; 11)	-0.74 (-3.13, 1.66)	0.55	3491 (3480; 11)	-2.10 (-9.65, 5.44)	0.58	3492 (3481; 11)	-0.12 (-3.96, 3.72)	0.95
24 years	2691 (2681; 10)	-1.53 (-4.55, 1.48)	0.32	2693 (2683; 10)	-5.80 (-15.19, 3.60)	0.23	2693 (2683; 10)	-1.18 (-5.12, 2.77)	0.56

BMI = body mass index; CI = confidence interval; cLoF = complete loss of function; GoF = gain of function; LoF = loss of function; pLoF = partial loss of function; SD = standard deviation; WT = wild-type. [1]N represents the total sample size in each analysis with numbers in brackets representing the number of individuals in the reference group (i.e., non-LoF carriers with synonymous, common variations or no LoF mutation and individuals with GoF mutations) and individuals carrying WT-like mutations, respectively. [2]Estimates represent the change in each anthropometric trait (units in table) in carriers vs. the reference group (e.g., non-LoF carriers with synonymous, common variations, no LoF mutation or GoF mutations). Analyses were linear regression models adjusted for sex, with p-values uncorrected for multiple comparisons.

Supplementary Table 8. Summary of measurements included in and model fit for BMI (kg/m^2) trajectories (N=5716).

	Summary of Measurements				Model fit for BMI trajectories		
	**Number of participants with at least one measure of BMI**[1]	**Total number of measures**	**Median (IQR) measures per participant**	**Mean (SD) predicted intercept and slopes**[2]	**Mean predicted BMI in kg/m^2 (SD)**[3]	**Mean observed BMI in kg/m^2 (SD)**	**Mean difference between observed and predicted BMI in kg/m^2 (95% CI)**[4]
Overall	5716	45622	9 (8 to 9)	-	-	-	-
18 months	646	646	1 (1 to 1)	17.04 (1.23)	17.03 (1.23)	17.12 (1.32)	0.09 (-0.85, 1.03)
18 months – 3.5 years	691	2875	5 (4 to 5)	-0.28 (0.28)	16.95 (1.27)	16.75 (1.37)	-0.01 (-0.91, 0.89)
3.5 – 5 years	652	1270	2 (2 to 3)	-0.20 (0.33)	16.26 (1.38)	16.27 (1.45)	0.01 (-0.76, 0.78)
5 – 8 years	4081	4281	1 (1 to 1)	0.61 (0.54)	16.17 (1.77)	16.13 (1.93)	-0.04 (-0.81, 0.72)
8 – 15 years	5695	32361	7 (6 to 7)	0.67 (0.32)	18.74 (3.25)	18.77 (3.37)	0.01 (-1.33, 1.34)
15 – 18 years	3841	4835	1 (1 to 2)	0.52 (0.97)	23.67 (5.63)	22.39 (3.93)	-0.02 (-1.41, 1.37)

BMI = body mass index; CI = confidence interval; IQR = inter-quartile range; SD = standard deviation

[1]Individuals who had full data on BMI and were in the sequence set. [2]The data at 18 months relate to predictions from the multilevel model at exactly 1.5 years (i.e., the intercept). [3]The data at 18 months relate to the BMI measurement carried out at mean age 1.53 years. [4]Range within which 95% of the differences between observed BMI measurements and those predicted by the multi-level model lie.

Supplementary Table 9. Summary of measurements included in and model fit for weight (kg) trajectories (N=5716).

	Summary of Measurements				Model fit for weight trajectories		
	Number of participants with at least one measure of weight[1]	Total number of measures	Median (IQR) measures per participant	Mean (SD) predicted intercept and slopes[2]	Mean predicted weight in kg (SD)[3]	Mean observed weight in kg (SD)	Mean difference between observed and predicted weight in kg (SD)[4]
Overall	5716	53083	10 (9 to 10)	-	-	-	-
Birth	5354	5354	1 (1 to 1)	3.51 (0.18)	3.51 (0.18)	3.43 (0.54)	-0.08 (-0.94, 0.78)
Birth – 12 months	717	6913	2 (2 to 3)	6.90 (0.87)	7.94 (1.92)	8.41 (1.67)	0.48 (-0.73, 1.68)
12 months – 8 years	4168	8792	7 (1 to 8)	2.28 (0.63)	19.64 (6.34)	19.73 (6.52)	0.08 (-1.58, 1.74)
8 – 15 years	5696	32541	7 (6 to 7)	4.69 (1.25)	42.42 (12.93)	42.51 (13.38)	-0.02 (-4.40, 4.37)
15 – 18 years	3843	4837	1 (1 to 2)	2.88 (2.84)	72.58 (20.40)	65.79 (13.24)	0.04 (-4.83, 4.91)

CI = confidence interval; IQR = inter-quartile range; SD = standard deviation.
[1]Individuals who had full data on weight and were in the sequence set. [2]The data at birth relate to predictions from the multilevel model at birth (i.e., the intercept). [3]The data at birth relate to the weight measurement carried out at birth. [4]Range within which 95% of the differences between observed weight measurements and those predicted by the multi-level model lie.

Supplementary Table 10. Summary of measurements included in and model fit for height (cm) trajectories (N=5716).

	**Summary of Measurements**				**Model fit for height trajectories**		
	**Number of participants with at least one measure of height[1]**	**Total number of measures**	**Median (IQR) measures per participant**	**Mean (SD) predicted intercept and slopes[2]**	**Mean predicted height in cm (SD)[3]**	**Mean observed height in cm (SD)**	**Mean difference between observed and predicted height in cm (SD)[4]**
Overall	5716	45962	9 (8 to 9)	-	-	-	-
18 months	647	647	1 (1 to 1)	82.77 (2.64)	82.77 (2.64)	81.69 (2.72)	-1.08 (-2.68, 0.52)
18 months – 5 years	692	4149	6 (6 to 7)	7.72 (0.69)	88.79 (11.11)	94.48 (9.12)	0.18 (-1.81, 2.16)
5 – 15 years	5704	36971	7 (6 to 8)	5.71 (0.47)	145.78 (14.90)	145.94 (15.32)	0.09 (-4.04, 4.23)
15 – 18 years	3844	4842	1 (1 to 2)	-4.69 (4.25)	159.91 (26.51)	171.26 (9.16)	-0.95 (-7.60, 5.70)

CI = confidence interval; IQR = inter-quartile range; SD = standard deviation.
[1]Individuals who had full data on height and were in the sequence set. [2]The data at 18 months relate to predictions from the multilevel model at exactly 1.5 years (i.e., the intercept). [3]The data at 18 months relate to the height measurement carried out at age 1.53 years.
[4]Range within which 95% of the differences between observed height measurements and those predicted by the multi-level model lie.

Supplementary Table 11. Association between *MC4R* LoF of cAMP accumulation with predicted weight trajectory between the ages at birth and 18 years using linear spline multi-level models (N=5716).

**Intercept and slopes**	**Mean weight trajectory (95% CI) in reference group[1]**	**Difference in the intercept and slopes between ages with *MC4R* LoF mutation[2]**	
		**Estimate (95% CI)**	**P-value**
Birth (kg)	3.44 (3.42, 3.47)	0.18 (-0.10, 0.47)	0.21
Change between birth – 12 months (kg/year)	6.50 (6.39, 6.61)	1.14 (-0.81, 3.09)	0.25
Change between 12 months – 8 years (kg/year)	2.39 (2.36, 2.42)	0.84 (0.40, 1.28)	1.63×10^{-04}
Change between 8 – 15 years (kg/year)	4.72 (4.66, 4.77)	1.33 (0.66, 1.99)	9.62×10^{-05}
Change between 15 – 18 years (kg/year)	1.11 (0.98, 1.24)	0.43 (-1.37, 2.23)	0.64

cLOF = complete loss of function; CI = confidence interval; GoF = gain of function; LoF = loss of function; pLoF = partial loss of function; WT-like = wild-type like.

[1]The reference group included all individuals with synonymous, common variations or no LoF mutation and individuals with GoF or WT-like mutations – the "non-LoF carriers". [2]Estimates represent the change in the intercept (kg) and slopes (kg/year) of weight in carriers vs. non-carriers of MC4R LoF mutations (i.e., individuals with synonymous, common variations or no LoF mutations and individuals with WT-like and GoF mutations). Linear spline multi-level models were adjusted for sex and p-values were not corrected for multiple comparisons.

Supplementary Table 12. Association between *MC4R* LoF of cAMP accumulation with predicted BMI trajectory between the ages of 18 months and 18 years using linear spline multi-level models (N=5716).

Intercept and slopes	Mean BMI trajectory (95% CI) in reference group[1]	Difference in the intercept and slopes between ages with *MC4R* LoF mutation[2]	
		Estimate (95% CI)	P-value
18 months (kg/m^2)	16.84 (16.72, 16.96)	0.81 (-1.39, 3.00)	0.47
Change between 18 months – 3.5 years (kg/m^2/year)	-0.23 (-0.27, -0.20)	0.13 (-0.46, 0.71)	0.67
Change between 3.5 – 5 years (kg/m^2/year)	-0.20 (-0.27, -0.13)	0.75 (-0.43, 1.94)	0.21
Change between 5 – 8 years (kg/m^2/year)	0.65 (0.63, 0.68)	0.39 (0.03, 0.75)	0.04
Change between 8 – 15 years (kg/m^2/year)	0.71 (0.70, 0.73)	0.12 (-0.08, 0.32)	0.25
Change between 15 – 18 years (kg/m^2/year)	0.28 (0.20, 0.36)	0.57 (-0.61, 1.74)	0.34

BMI = body mass index; CI = confidence interval; cLOF = complete loss of function; GoF = gain of function; LoF = loss of function; pLOF = partial loss of function.

[1]The reference group included all individuals with synonymous, common variations or no LoF mutation and individuals with GoF or WT-like mutations – the "non-LoF carriers". [2]Estimates represent the change in the intercept (kg/m^2) and slopes (kg/m^2/year) of BMI in carriers vs. non-carriers of MC4R LoF mutations (i.e., individuals with synonymous, common variations or no LoF mutations and individuals with WT-like and GoF mutations). Linear spline multi-level models were adjusted for sex and p-values were not corrected for multiple comparisons.

Supplementary Table 13. Association between *MC4R* LoF of cAMP accumulation with predicted height trajectory between the ages of 18 months and 18 years using linear spline multi-level models (N=5716).

**Intercept and slopes**	**Mean height trajectory (95% CI) in reference group[1]**	**Difference in the intercept and slopes between ages with *MC4R* LoF mutation[2]**	
		**Estimate (95% CI)**	**P-value**
18 months (cm)	81.90 (81.70, 82.11)	2.11 (-1.50, 5.73)	0.25
Change between 18 months – 5 years (cm/year)	7.79 (7.74, 7.84)	0.48 (-0.29, 1.26)	0.23
Change between 5 – 15 years (cm/year)	5.50 (5.48, 5.53)	0.07 (-0.23, 0.38)	0.64
Change between 15 – 18 years (cm/year)	-8.14 (-8.34, -7.95)	-2.27 (-5.12, 0.57)	0.12

cLoF = complete loss of function; CI = confidence interval; GoF = gain of function; LoF = loss of function; pLoF = partial loss of function; WT-like = wild-type like.

[1]The reference group included all individuals with synonymous, common variations or no LoF mutation and individuals with GoF or WT-like mutations – the "non-LoF carriers". [2]Estimates represent the change in the intercept (cm) and slopes (cm/year) of height in carriers vs. non-carriers of MC4R LoF mutations (i.e., individuals with synonymous, common variations or no LoF mutations and individuals with WT-like and GoF mutations). Linear spline multi-level models were adjusted for sex and p-values were not corrected for multiple comparisons.

Supplementary Table 14. Age-specific associations between *MC4R* LoF of β-arrestin-2 coupling and BMI, weight and height.

Age	BMI (kg/m²)			Weight (kg)			Height (kg)		
	N (ref, pLoF, cLoF)[1]	Effect estimate (95% CI)[2]	P	N (ref, pLoF, cLoF)[1]	Effect estimate (95% CI)[2]	P	N (ref, pLoF, cLoF)[1]	Effect estimate (95% CI)[2]	P
Birth				5354 (5341; 8; 5)	0.25 (-0.04, 0.54)	0.09			
4 months	967 (965; 2; 0)	-0.70 (-2.78, 1.39)	0.51	1034 (1031; 3; 0)	0.26 (-0.61, 1.13)	0.56	970 (968; 2; 0)	0.87 (-2.50, 4.25)	0.61
8 months	3307 (3301; 6; 0)	-0.56 (-1.79, 0.68)	0.38	3690 (3683; 6; 1)	0.02 (-0.71, 0.76)	0.95	3375 (3369; 6; 0)	0.08 (-2.03, 2.20)	0.94
12 months	1370 (1368; 2; 0)	1.11 (-0.94, 3.16)	0.29	1613 (1609; 3; 1)	1.77 (0.66, 2.88)	0.002	1386 (1384; 2; 0)	1.04 (-2.80, 4.88)	0.60
18 months	2719 (2713; 4; 2)	1.16 (-0.06, 2.38)	0.06	2905 (2899; 4; 2)	1.36 (0.36, 2.37)	0.01	2797 (2791; 4; 2)	1.66 (-0.81, 4.13)	0.19
2 years	891 (889; 2; 0)	0.73 (-1.26, 2.71)	0.47	925 (923; 2; 0)	1.20 (-0.79, 3.19)	0.24	913 (911; 2; 0)	2.15 (-2.20, 6.49)	0.33
2.5 years	673 (671; 2; 0)	-0.09 (-2.01, 1.82)	0.92	698 (696; 2; 0)	0.79 (-1.48, 3.06)	0.50	681 (679; 2; 0)	3.07 (-1.46, 7.59)	0.18
3 years	1052 (1050; 2; 0)	-0.09 (-2.10, 1.93)	0.93	1081 (1079; 2; 0)	0.61 (-1.86, 3.08)	0.63	1097 (1095; 2; 0)	2.37 (-2.66, 7.39)	0.36
3.5 years	2443 (2436; 5; 2)	0.23 (-0.78, 1.24)	0.65	2522 (2515; 5; 2)	1.42 (0.03, 2.82)	0.04	2527 (2518; 6; 3)	4.44 (1.99, 6.90)	$3.92x10^{-04}$
4 years	1111 (1109; 2; 0)	0.03 (-2.00, 2.06)	0.98	1131 (1129; 2; 0)	0.82 (-2.19, 3.83)	0.59	1154 (1152; 2; 0)	2.44 (-3.19, 8.07)	0.40
5 years	1284 (1281; 2; 1)	0.77 (-1.23, 2.77)	0.45	1321 (1318; 2; 1)	2.55 (-0.67, 5.77)	0.12	1383 (1380; 2; 1)	3.73 (-2.28, 9.73)	0.22
8 years	5243 (5232; 6; 5)	3.42 (2.23, 4.61)	$1.86x10^{-08}$	5243 (5232; 6; 5)	8.19 (5.45, 10.92)	$4.75x10^{-09}$	5248 (5237; 6; 5)	5.36 (1.99, 8.72)	0.002
9 years	4452 (4441; 6; 5)	4.27 (2.89, 5.66)	$1.37x10^{-09}$	4560 (4549; 6; 5)	11.56 (8.18, 14.93)	$2.10x10^{-11}$	4715 (4704; 6; 5)	6.77 (3.34, 10.21)	$1.13x10^{-04}$
10 years	5378 (5366; 7; 5)	4.54 (2.94, 6.13)	$2.42x10^{-08}$	5412 (5400; 7; 5)	13.94 (9.81, 18.08)	$4.32x10^{-11}$	5381 (5369; 7; 5)	7.85 (4.27, 11.43)	$1.76x10^{-05}$
11 years	5050 (5038; 8; 4)	3.66 (1.95, 5.38)	$2.87x10^{-05}$	5084 (5072; 8; 4)	11.38 (6.71, 16.04)	$1.79x10^{-06}$	5059 (5047; 8; 4)	5.98 (2.21, 9.74)	0.002
12 years	4929 (4918; 7; 4)	4.60 (2.64, 6.56)	$4.41x10^{-06}$	4934 (4923; 7; 4)	16.85 (11.07, 22.63)	$1.15x10^{-08}$	4931 (4920; 7; 4)	9.12 (4.87, 13.37)	$2.64x10^{-05}$
13 years	4650 (4640; 6; 4)	5.78 (3.67, 7.90)	$9.13x10^{-08}$	4650 (4640; 6; 4)	19.60 (13.00, 26.21)	$6.24x10^{-09}$	4695 (4685; 6; 4)	7.58 (2.82, 12.33)	0.002
14 years	4352 (4343; 5; 4)	4.51 (2.27, 6.75)	$7.97x10^{-05}$	4352 (4343; 6; 4)	18.82 (11.49, 26.15)	$5.03x10^{-07}$	4357 (4348; 5; 4)	9.04 (4.03, 14.05)	$4.10x10^{-04}$
15 years	3866 (3856; 6; 4)	4.99 (2.87, 7.11)	$3.95x10^{-06}$	3866 (3856; 6; 4)	20.78 (13.75, 27.81)	$7.38x10^{-09}$	3872 (3862; 6; 4)	7.60 (3.34, 11.86)	$4.69x10^{-04}$
18 years	3499 (3492; 4; 3)	5.23 (2.23, 8.23)	$6.43x10^{-04}$	3501 (3494; 4; 3)	21.76 (12.31, 31.22)	$6.63x10^{-06}$	3502 (3495; 4; 3)	6.46 (1.64, 11.27)	0.01
24 years	2695 (2693; 1; 1)	0.94 (-5.79, 7.67)	0.79	2697 (2695; 1; 1)	11.92 (-9.05, 32.89)	0.27	2697 (2695; 1; 1)	9.42 (0.61, 18.22)	0.04

BMI = body mass index; CI = confidence interval; cLoF = complete loss of function; GoF = gain of function; LoF = loss of function; pLoF = partial loss of function; SD = standard deviation; WT = wild-type. [1]N represents the total sample size in each analysis with numbers in brackets representing the number of individuals in the reference group (i.e., individuals with synonymous, common variations or no LoF mutation and individuals with GoF or WT-like mutations – the "non-LoF carriers"), and individuals with pLoF or cLoF mutations, respectively. [2]Estimates represent the change in each anthropometric trait (units in table) in carriers vs. non-carriers of MC4R LoF mutations. Analyses were linear regression models adjusted for sex, with p-values uncorrected for multiple comparisons.

Supplementary Table 15. Age-specific associations between *MC4R* LoF of β-arrestin-2 coupling and WHR, fat mass and lean mass.

Age	Fat mass (kg)			Lean mass(kg)			WHR		
	N (ref, pLoF, cLoF)[1]	Effect estimate (95% CI)[2]	P	N (ref, pLoF, cLoF)[1]	Effect estimate (95% CI)[2]	P	N (ref, pLoF, cLoF)[1]	Effect estimate (95% CI)[2]	P
8 years							5078 (5067; 6; 5)	-0.003 (-0.03, 0.02)	0.79
10 years	5109 (5097; 7; 5)	9.47 (6.70, 12.23)	$2.12x10^{-11}$	5109 (5097; 7; 5)	3.88 (2.15, 5.62)	$1.19x10^{-05}$	5339 (5327; 7; 5)	0.03 (0.01, 0.06)	0.02
12 years	4874 (4863; 7; 4)	10.65 (6.78, 14.52)	$7.27x10^{-08}$	4874 (4863; 7; 4)	5.55 (2.98, 8.11)	$2.31x10^{-05}$	4926 (4915; 7; 4)	0.04 (0.01, 0.07)	0.01
14 years	4295 (4286; 5; 4)	12.21 (7.28, 17.15)	$1.27x10^{-06}$	4295 (4286; 5; 4)	5.93 (2.09, 9.77)	0.002			
15 years	3750 (3740; 6; 4)	14.42 (9.36, 19.47)	$2.40x10^{-08}$	3750 (3740; 6; 4)	5.17 (1.75, 8.59)	0.003			
18 years	3408 (3401; 4; 3)	17.73 (10.69, 24.78)	$8.28x10^{-07}$	3408 (3401; 4; 3)	2.71 (-1.22, 6.65)	0.18			
24 years	2631 (2629; 1; 1)	11.90 (-2.49, 26.29)	0.11	2631 (2629; 1; 1)	-0.45 (-9.21, 8.32)	0.92	2692 (2690; 1; 1)	0.04 (-0.04, 0.12)	0.35

CI = confidence interval; cLoF = complete loss of function; GoF = gain of function; LoF = loss of function; pLoF = partial loss of function; SD = standard deviation; WHR = waist-hip ratio WT = wild-type

[1]N represents the total sample size in each analysis with numbers in brackets representing the number of individuals in the reference group (i.e., individuals with synonymous, common variations or no LoF mutations and individuals with GoF or WT-like mutations – the "non-LoF carriers"), and individuals with pLoF or cLoF mutations, respectively. [2]Estimates represent the change in each anthropometric trait (units in table) in carriers vs. non-carriers of MC4R LoF mutations. Analyses were linear regression models adjusted for sex, with p-values uncorrected for multiple comparisons.

Supplementary Table 16. Age-specific associations between the weighted genome-wide polygenic risk score and BMI.

Age of BMI measurement	N	Effect estimate (95% CI)[1]	P-value
4 months	903	0.30 (-0.02, 0.63)	0.06
8 months	3012	0.13 (-0.06, 0.31)	0.18
12 months	1263	0.17 (-0.09, 0.44)	0.20
18 months	2478	0.11 (-0.10, 0.31)	0.30
2 years	810	0.18 (-0.14, 0.51)	0.27
2.5 years	629	-0.02 (-0.35, 0.31)	0.91
3 years	968	0.28 (-0.02, 0.57)	0.07
3.5 years	2229	0.39 (0.20, 0.58)	$6.79x10^{-05}$
4 years	1010	0.38 (0.09, 0.68)	0.01
5 years	1182	0.59 (0.27, 0.91)	$3.54x10^{-05}$
8 years	4752	1.06 (0.87, 1.25)	$5.34x10^{-27}$
9 years	4050	1.22 (0.98, 1.46)	$5.90x10^{-23}$
10 years	4868	1.64 (1.38, 1.90)	$5.96x10^{-34}$
11 years	4579	1.81 (1.52, 2.10)	$1.76x10^{-33}$
12 years	4474	1.89 (1.56, 2.22)	$2.94x10^{-29}$
13 years	4208	1.99 (1.64, 2.34)	$2.13x10^{-28}$
14 years	3950	1.80 (1.43, 2.16)	$3.71x10^{-22}$
15 years	3510	2.05 (1.67, 2.43)	$3.17x10^{-25}$
18 years	3164	2.64 (2.17, 3.10)	$3.28x10^{-28}$
24 years	2442	3.10 (2.46, 3.73)	$2.70x10^{-21}$

BMI = body mass index; CI = confidence interval

[1]Estimates represent the change in BMI (kg/m^2) between the top 10th percentile vs. lower 90th percentile of the weighted genome-wide polygenic risk score, restricted to only those in the sequence set. Analyses were linear regression models adjusted for sex, with p-values uncorrected for multiple comparisons.

Supplementary Table 17. Summary of measurements included in and model fit for BMI (kg/m^2) trajectories (N=5162).

	**Summary of Measurements**				**Model fit for BMI trajectories**		
	**Number of participants with at least one measure of BMI[1]**	**Total number of measures**	**Median (IQR) measures per participant**	**Mean (SD) predicted intercept and slopes[2]**	**Mean predicted BMI in kg/m^2 (SD)[3]**	**Mean observed BMI in kg/m^2 (SD)**	**Mean difference between observed and predicted BMI in kg/m^2 (95% CI)[4]**
Overall	5162	41445	9 (8 to 9)	-	-	-	-
18 months	596	596	1 (1 to 1)	17.08 (1.24)	17.08 (1.24)	17.15 (1.33)	0.07 (-0.70, 0.83)
18 months – 3.5 years	638	2667	5 (4 to 5)	-0.31 (0.38)	16.97 (1.31)	16.78 (1.38)	-0.01 (-0.82, 0.80)
3.5 – 5 years	602	1167	2 (2 to 3)	-0.34 (0.22)	16.24 (1.39)	16.30 (1.48)	0.06 (-0.67, 0.78)
5 – 8 years	3557	3735	1 (1 to 1)	0.28 (0.55)	16.24 (1.92)	16.13 (1.92)	-0.11 (-1.06, 0.84)
8 – 15 years	5144	29498	7 (6 to 7)	0.67 (0.31)	18.70 (3.23)	18.74 (3.36)	0.01 (-1.44, 1.46)
15 – 18 years	3475	4378	1 (1 to 2)	0.58 (0.49)	23.83 (4.62)	22.39 (3.90)	-0.01 (-1.51, 1.49)

BMI = body mass index; CI = confidence interval; IQR = inter-quartile range; SD = standard deviation.
[1]Individuals who had full data on BMI and were in the sequence set. [2]The data at 18 months relate to predictions from the multilevel model at exactly 1.5 years (i.e., the intercept). [3]The data at 18 months relate to the BMI measurement carried out at a mean age of 1.53 years.

Supplementary Table 18. Association between the weighted genome-wide polygenic risk score (lower 90th and upper 10th percentiles) with predicted BMI trajectory between the ages of 18 months and 18 years in the sequence set using linear spline multi-level models (N=5162).

Intercept and slopes	Mean BMI trajectory (95% CI) in reference group[1]	Difference in the intercept and slopes between ages with the genome-wide polygenic risk score (upper 10th vs. lower 90th percentile)[2]	
		Estimate (95% CI)	P-value
18 months (kg/m^2)	16.63 (16.49, 16.77)	-0.09 (-0.39, 0.21)	0.55
Change between 18 – 3.5 years (kg/m^2/year)	-0.26 (-0.31, -0.20)	0.10 (-0.02, 0.22)	0.10
Change between 3.5 – 5 years (kg/m^2/year)	-0.34 (-0.40, -0.27)	0.19 (0.06, 0.32)	0.01
Change between 5 – 8 years (kg/m^2/year)	0.28 (0.23, 0.33)	0.31 (0.21, 0.41)	$3.22\text{x}10^{-09}$
Change between 8 – 15 years (kg/m^2/year)	0.71 (0.69, 0.72)	0.14 (0.10, 0.18)	$2.74\text{x}10^{-14}$
Change between 15 – 18 years (kg/m^2/year)	0.49 (0.45, 0.53)	0.10 (0.003, 0.20)	0.04

BMI = body mass index; CI = confidence interval

[1]The reference group included individuals in the lower 90th percentile of the weighted genome-wide polygenic risk score. [2] Linear spline multi-level models were adjusted for sex and p-values were not corrected for multiple comparisons.

Supplementary Table 19. Age-specific associations between *MC4R* LoF of cAMP accumulation and BMI, adjusting for the weighted genome-wide polygenic risk score.

Age	BMI (kg/m²)		
	N[1]	Effect estimate (95% CI)[2]	P
Birth			
4 months	903	-0.25 (-3.22, 2.72)	0.87
8 months	3010	0.08 (-1.06, 1.23)	0.89
12 months	1261	1.59 (-0.46, 3.64)	0.13
18 months	2477	0.85 (-0.63, 2.33)	0.26
2 years	810	-0.32 (-3.13, 2.48)	0.82
2.5 years	629	-0.63 (-3.34, 2.07)	0.65
3 years	969	-0.54 (-3.39, 2.30)	0.71
3.5 years	2228	0.07 (-1.02, 1.15)	0.91
4 years	1010	-0.61 (-2.61, 1.39)	0.55
5 years	1181	0.35 (-2.06, 2.76)	0.78
8 years	4752	1.79 (0.65, 2.94)	0.002
9 years	4050	2.58 (1.19, 3.97)	$2.76x10^{-04}$
10 years	4868	2.77 (1.25, 4.28)	$3.40x10^{-04}$
11 years	4579	2.34 (0.78, 3.90)	0.003
12 years	4474	2.77 (0.91, 4.64)	0.004
13 years	4208	3.77 (1.75, 5.79)	$2.50x10^{-04}$
14 years	3950	2.21 (0.08, 4.35)	0.04
15 years	3510	3.74 (1.72, 5.77)	$3.01x10^{-04}$
18 years	3164	4.46 (1.64, 7.28)	0.002
24 years	2442	-0.34 (-6.70, 6.01)	0.92

BMI = body mass index; CI = confidence interval; cLoF = complete loss of function; GoF = gain of function; LoF = loss of function; pLoF = partial loss of function; SD = standard deviation; WT = wild-type.

[1]N represents the total sample size in each analysis. [2]Estimates represent the change in each anthropometric trait (units in table) in carriers (i.e., individuals with pLoF or cLoF mutations) vs. non-carriers (i.e., individuals with synonymous, common variations or no LoF mutation, and individuals with GoF or WT-like mutations) of MC4R LoF mutations, after adjusting for sex and the weighted genome-wide polygenic risk score, with p-values uncorrected for multiple comparisons.

Supplementary Table 20. Primers used for Human *MC4R* Sequencing.

Primer name	Sequence (5'-3')	Chromosome 18 position[1]		Strand
MC4R Exon_Forward	GGGGGACACTGGAATTCTCC	60372357	60372376	Negative
MC4R Exon_Reverse	ACCCTACACGGAAGAGAAAGC	60371247	60371267	Positive
MC4R_Sequencing_1_Forward	ACTGGAATTCTCCTGCCAGC	60372350	60372369	Negative
MC4R_Sequencing_1_Reverse	CCAACAAGCTGATGACACCC	60372169	60372188	Positive
MC4R_Sequencing_2_Forward	TGACTCTGGGTGTCATCAGC	60372176	60372195	Negative
MC4R_Sequencing_2_Reverse	TGGATGCAAGCAAGGAGCTA	60371941	60371960	Positive
MC4R_Sequencing_3_Forward	ACTCGGTGATCTGTAGCTCCT	60371953	60371973	Negative
MC4R_Sequencing_3_Reverse	GAAGCCATGAGAGCCAGCAT	60371721	60371740	Positive
MC4R_Sequencing_4_Forward	TGTTCTTCACCATGCTGGCT	60371732	60371751	Negative
MC4R_Sequencing_4_Reverse	GTGAGACATGAAGCACACACA	60371501	60371521	Negative
MC4R_Sequencing_5_Reverse	CACGGAAGAGAAAGCTGTTGC	60371253	60371273	Positive

[1]*Assembly: GRCh38p13*
